# The Guyana Diabetes and Foot Care Project: A Complex Quality Improvement Intervention to Decrease Diabetes-Related Major Lower Extremity Amputations and Improve Diabetes Care in a Lower-Middle-Income Country

**DOI:** 10.1371/journal.pmed.1001814

**Published:** 2015-04-21

**Authors:** Julia Lowe, R. Gary Sibbald, Nashwah Y. Taha, Gerald Lebovic, Carlos Martin, Indira Bhoj, Rolinda Kirton, Brian Ostrow, the Guyana Diabetes and Foot Care Project Team

**Affiliations:** 1 Division of Endocrinology, University of Toronto, Toronto, Ontario, Canada; 2 Dalla Lana School of Public Health, University of Toronto, Toronto, Ontario, Canada; 3 Applied Health Research Centre (AHRC), St. Michael's Hospital University of Toronto, Toronto, Ontario, Canada; 4 Georgetown Public Hospital Corporation, Ministry of Health Guyana, Georgetown, Guyana; 5 Guyana Diabetes and Foot Care Project, Georgetown Public Hospital Corporation, Georgetown, Guyana; 6 Department of Surgery, University of Toronto, Toronto, Ontario, Canada

## Abstract

Julia Lowe and colleagues describe the Guyana Diabetes Foot Care Project, a quality improvement intervention for improving diabetes care in a low-income setting.

**Summary Points**

- Type 2 diabetes is the fourth leading cause of death and affects 15.5% of the adult population in Guyana, South America.
- Preintervention, 41.4% of individuals with diabetic foot complications experienced major lower extremity amputation at the national referral hospital.
- A complex, interprofessional quality improvement intervention to improve diabetes and foot care was rolled out in two phases between 2008–2013.
- We report the experience from this unique nationwide intervention, with a national referral hospital prototype (phase 1) regionalized to six administrative regions in Guyana comprising 89% of the population (phase 2).

## The Challenge

The long-term complications and ill effects of Type 2 diabetes are preventable [1], but systems to ensure this prevention occurs have been poorly implemented [2,3], especially in low- and middle-income countries (LMIC). Recent diabetes-specific quality improvement (QI) research has demonstrated that interventions targeting chronic disease management systems are most likely to improve diabetes care, and interventions targeting health care professionals are beneficial, if baseline glycosylated hemoglobin (HbA1c) control is poor [4]. A systematic review identified three priority levels of interventions for preventing and treating diabetes and its complications in LMIC [5]. Level 1 interventions are considered cost saving and highly feasible. They are (i) foot care in people with a high risk of ulcers, (ii) blood pressure (BP) control (<160/95 mm Hg), and (iii) glycemic control (HbA1c < 9%; 75 mmol/mol). These interventions were targeted in the Guyana Diabetes and Foot Care Project (GDFP).

## The Setting

Guyana is a multiethnic country with people of South Asian, African, and Amerindian origin, all of whom are at high risk for developing diabetes. The third poorest country in South America, [6] Guyana suffers a net loss of population, especially health care professionals [7]. Despite this, the government provides free universal health care through a multilevel healthcare system (health posts, health centres, regional hospitals, and a national referral and teaching hospital). Coverage is provided for an estimated 80% of the population and includes free medications from the national formulary [8].

In Guyana, it was estimated that diabetes was the fourth leading cause of death in 2008 [9], and the adult prevalence had risen to 15.5% by 2011 [10]. At Georgetown Public Hospital Corporation (GPHC), the national referral and teaching hospital, complications of diabetic foot problems were the most common cause of admission to the surgical ward until 2008 [11], and 42% of these patients ended up with a lower extremity amputation. Local health care leaders were keen to change this and partnered with Canadian surgeons and wound and foot care experts who confirmed these findings during exploratory visits. In addition, they identified lack of care coordination and a systems approach (practice in silos, no foot ulcer prevention, no plantar pressure redistribution, and narrow spectrum and missed antimicrobial doses) as factors amenable to change. They also found staff demoralized by the high burden of disease (30% inpatient population), prolonged stays, and frequent readmissions with poor patient outcomes. Surgical debridement often took place in poorly lit and unsanitary wards, with inappropriate use of amputation.

## The Guyana Diabetes and Foot Care Project: 2008–2013

The time course of the two phases is shown in Table 1. The challenges facing the Guyanese health care system were identified and the model of care developed using multilevel knowledge-to-action (K2A) cycles [12].

**Table 1:** Time course of the Guyana Diabetes and Foot Care Project.

GUYANA DIABETES AND FOOT CARE PROJECT							
PHASES		Phase 1—CCF Project No 530-0272-G015		Phase 2—Project No. S064802			
Lead organization		Georgetown Public Hospital Corporation		Canadian Association of General Surgeons (CAGS)			
FUNDING		Can$97,460		Can$498,925			
		GOVERNMENT OF CANADA—CANADIAN INTERNATIONAL DEVELOPMENT AGENCY (CIDA)					
Item	2007	2008	2009	March 2010	2011	2012	28 Feb 2013
**Goal**	• Needs assessment	“Improve health of persons with diabetes in Guyana by developing an enhanced diabetic foot program at the Georgetown Public Hospital Corporation”		“Regionalization of diabetes and diabetic foot care in 6/10 administrative regions comprising 90% [of the] population in Guyana”			
	• Funding application						
**Facility development**		Diabetic Foot Care Centre (DFC) at GPHC		Seven regional diabetic foot care centres were established and staffed, but three were completed only in the last 6 months.			
		Achieved Centre of Excellence status Dec 2009					
**Key opinion leader (KOL) training**		Interprofessional (MD, nurse, and rehab specialist) teams in two cohorts—eight people at the International Interprofessional Wound Care Course (IIWCC)		• Three cohorts at IIWCC comprising eight people—total in phase 1 and phase 2 = 16 (14 completed, nine retained)			
				• Online diabetes educator course—10 people			
**Iterative continuous medical education (CME), best practice workshops, and preceptorships**		• Diabetic foot care (3-day workshops)		Diabetes, hypertension, and dyslipidemia care (3-day workshops) in addition to diabetic foot care—delivered by KOL team			
		• Additional 5-day course for pregraduate rehabilitation assistants					
**Health care professionals (HCP) trained**		65 trained at GPHC		275 trained at 97 facilities			
**Simplified 60-second diabetic foot screening tool**		• Developed and introduced screen in July 2008		• 3,452 screens in regions, 65% at high risk			
		• 1,226 patients screened at GPHC, 48% at high risk		• Screen validated in June 2012			
**Care innovations**		• Comprehensive foot assessments with Doppler and skin temps		HbA1c testing began in April 2010—new to Guyana			
		• Care based on context-specific best practices, conservative debridement		• Mean HbA1c of 4,588 people = 8.6% (70.5 mmol/mol)			
		• Plantar pressure reduction for patients with foot ulcers		• 37.3% tested had HbA1c > 9% (75 mmol/mol)			
**Mentoring visits by Canadian experts**		Four visits over 18 months + additional chiropodist visit to train rehab assistants		Six visits over 3 years			
**Integration into MoH standard operating procedure**		Simplified 60-second foot screening tool approved		• KOL team retained as trainers			
				• Identification and approval of indicators for data collection and development of data collection processes, tools, and human resources.			
				• Model approved in “Strategic Plan 2013–2020”			

The GDFP project costs were funded by the Canadian International Development Agency (CIDA). To avoid development of parallel unsustainable systems, clinical activities resided within the Guyanese public health system, and staff and supplies were funded by Ministry of Health (MoH). The phase 1 goal was to create health care changes to improve foot care in people with diabetes (PWD) and reduce diabetes-related lower extremity amputations (LEA) at GPHC. Guyanese key opinion leaders (KOLs) were trained using established programs such as the International Interprofessional Wound Care Course (IIWCC) [13]; this was supplemented by best practice seminars and preceptorships, along with enablers for practice built on the Wound Bed Preparation paradigm. Phase 2 concentrated on building capacity across the country, with regionalization of the foot care model and expansion to include KOL training through the Canadian Michener Institute’s International Diabetes Federation (IDF)–recognized diabetes educator course. Three-day training programs on improved management of diabetes, including diabetic foot and hypertension, were held in six administrative regions comprising 89% of the population. Suitable regional centres and training sites were identified in conjunction with the MoH, with budgets and work plans developed. A project database captured more complex project outcomes, with clerks appointed and trained in data entry at each centre. The project database combines HbA1c data from the high-performance liquid chromatography machine at GPHC with foot screening and diabetes data from regional foot centres using a unique patient identifier.

A Canadian endocrinologist (JL) and diabetes educators developed the training program to address diabetes, hypertension, and dyslipidemia based on Canadian and Caribbean guidelines. Workshops incorporated the basics of adult education, behavior change, and an introduction to health systems theory and were adapted to local conditions by KOLs before being delivered to multiprofessional groups of health care professionals from targeted regions. The Canadian team supervised the first workshop given by Guyanese KOLs to ensure consistency and quality.

The key steps in both phases 1 and 2 of the project are described below [12].

### 1. Key Knowledge Transfer Concepts

#### Prevention

Deployment of limited resources was achieved by using a clinical screening tool to identify loss of protective sensation. This simplified 60-second screening tool [14] identified patients at high risk of developing foot ulcers, who were then referred for more intensive surveillance, education on foot care, foot wear, glycemic control and smoking cessation, and debridement of calluses linked to the use of offloading devices [15]. A reliability study confirmed the utility of this tool, which was adopted by the MoH for use throughout Guyana.

#### Recognized best practices

Previous projects and a literature review identified existing tools suitable for education and skills training of local health care professionals [16]. These were modified by a Canadian wound-healing expert (RGS) and endocrinologist (JL). Absence and cost of wound care products used by high-income countries led to adaptation of wound care practices. In the absence of any foot specialists in Guyana, principles of plantar pressure redistribution therapy were taught to rehabilitation assistants and orthotic, prosthetic, and cast room technicians.

#### Use of adult-learning principles

Using the strategies shown in Box 1, we promoted and demonstrated interprofessional collaboration [17], with the education of Guyanese health care professionals carried out in interprofessional teams.

**Box 1. Use of Adult-Learning Principles**

- Iterative interprofessional best practice sessions (CMEs) presented by key opinion leaders in 3-day workshops:
  - Wound bed preparation with special emphasis on VIP: vascular supply, infection identification and treatment, and plantar pressure redistribution (Phases 1 and 2)
  - Management of diabetes and hypertension (Phase 2)
- Skills training in the clinic for comprehensive assessment of the foot and management of diabetes, dyslipidemia, and hypertension
- Preceptorships to model interprofessional communication, collaboration, and coordination for improved patient care and outcomes
- Provision of practice aids (enablers) and patient assessment tools
- On-job training and mentorship (reinforcing strategies)

## 2. Assess and Manage Barriers to Knowledge Use in Guyana—Box 2

**Box 2. Identified Barriers to Knowledge Use in Guyana**

- Assessment of patient and staff attitudes and beliefs:
  - Health care professionals were working in silos (only one rehabilitation assistant consult from surgery in 2 years).
  - No comprehensive assessment process for people admitted with diabetic foot ulcers
  - Nurses were demoralized as a result of the heavy burden of patient care and disease severity, without having any capacity to initiate change.
  - Medical officers in the weekly outpatient diabetes clinic were resistant to participate in project activities.
  - Patients presented late for treatment, with a poor level of diabetes knowledge.
  - Men are reluctant to come for diabetes screening, but, relatively, a higher percentage are admitted for foot complications.
  - Very poor footwear—open “rubber dinkys” or “flip-flops” with strap between first and second toes with no plantar pressure redistribution modalities (e.g., shoes, orthotics, and other plantar pressure redistribution devices)
- Absence of health care human resources—no chiropodists, podiatrists, endocrinologists, or vascular surgery capacity in Guyana
- Lack of equipment and resources—no dopplers or skin thermometers; no plantar pressure redistribution therapy; and no HbA1c testing in the public system or home glucose testing
- Frequent interruptions in the supply chain for drugs (e.g., metformin) and dressings provided through MoH
- Lack of funding—MoH is heavily dependent on foreign contributions to support health care. In 2007, 48% of total health care expenditures were from external sources, mostly designated for HIV care.

## 3. Implementation

Strategies were those identified as strongly or moderately effective by a systematic review of guideline implementation methods [18] and addressed issues for patients, health care professionals, hospital administration, and the government. Key interventions are detailed in Box 3.

**Box 3. Key Interventions**

- **Prevention:** A screening tool was developed and applied in the GPHC weekly outpatient diabetes clinic (population base of 2,000 patients). Inlow’s 60-second foot screening exam was modified to produce a Guyana-based 60-second screening tool for the high-risk diabetic foot. A single positive score on this test was considered indicative of high-risk status and resulted in referral to the newly created Diabetic Foot Centre for surveillance, foot care, and foot wear education and, if necessary, plantar pressure redistribution. This tool has been further modified as a screening for the high-risk diabetic foot: A 60-second tool (2014) Sibbald.
- **KOL Team:** The pivotal intervention to provide local leadership and ensure sustainability was the development of the interprofessional key opinion leader team. This is a development from the standard “train the trainer” model in that the KOL team is not only responsible for local education but also provides clinical leadership for system change.
- **Educational platform and training:** In phases 1 and 2, doctors, nurses, and rehabilitation specialists attended the previously established IIWCC at the University of Toronto, a 9-month course providing interactive, advanced education in all aspects of the care of chronic wounds, as well as in the principles of adult education. In phase 2 a diabetes educator course was held in Guyana under the auspices of the Michener Institute for Applied Health Sciences in Toronto.
- **Facility, staffing, and resources:** A space for the outpatient Diabetic Foot Centre (DFC) was assigned by GPHC administration, and the DFC was opened in July 2008. The unit is led by the KOL team and staffed by health care professionals trained in the project’s educational platform. High-risk patients referred from the diabetic outpatient clinic and elsewhere are assessed, educated, treated, and followed. A mandatory consult is required for all patients admitted with diabetic foot complications. The role of nursing to coordinate continuing care is supported and the critical need for rehabilitation assessment for pressure offloading emphasized. A clerk was hired to coordinate clinic scheduling and data entry. Computer and other office supplies were purchased and spreadsheets developed to track the patient population. In phase 2, staff in these and other community centres received additional education and skills training in management of blood pressure and hyperglycaemia.
- **Systems change:** Systems change was facilitated through networking with key stakeholders, the Ministry of Health, and key hospital GPHC. As project donor, CIDA cofacilitated important project-related discussions between the key partners. Memoranda of understanding were signed with all stakeholders.
- **Community awareness:** Community awareness was enhanced by the use of media such as television, including a Ministry of Health program called “Your Health, The Nation’s Wealth” that helped to increase awareness of diabetic foot complications by outlining signs of danger to the feet. MoH patient diabetes, hypertension, and foot care information posters and pamphlets were revised, edited, and made available throughout the public health system. Additional patient information handouts were developed as an adjunct to DFC care.

## 4. Monitor and Adjust the Intervention

Ten visits to Guyana by interprofessional Canadian experts re-enforced and enabled knowledge and practice. Initially led by the Canadian team, interprofessional best practices were transferred to leadership by the KOLs and fine-tuned during subsequent Canadian team visits.

## System Change

From its inception, the project recognized the necessity of sustaining benefits beyond its lifespan. Sustainability was planned by embedding all activities (e.g., training, clinical best practices, and facility renovations) inside the public system. System change was facilitated through networking with key stakeholders, including the Ministers of Health, hospital chief executive officers (CEOs), regional medical directors, and other officials. The MoH embraced the model, as shown by approval of all project assessment tools, indicators, guidelines and data spreadsheets, budgets and purchases of supplies, and the ongoing use of KOL teams in training medical, nursing, and rehabilitation staff. The KOL team is now under the management of the Institute of Health Sciences Education, which is responsible for postgraduate medical and health education in Guyana. Project innovations were included in the 2012 Service Level Agreements between the regions and MoH and the project model approved in the new “MOH Strategic Plan 2013–2020: Integrated Prevention and Control of Non-communicable Disease in Guyana” [19]. This plan recognises that the project uses the Pan American Health Organization (PAHO) chronic care model (CCM) and states its intention to “integrate CCM used in regional diabetes foot care project into ‘Wellness Centres of Excellence.’” This plan and the appointment of a coordinator for chronic noncommunicable diseases (CNCD) are extremely important developments and lay the groundwork for further collaboration.

## Outcomes

Fourteen key opinion leaders successfully completed the IIWCC, and 340 health care professionals from 97 facilities were trained. One centre of excellence and seven regional diabetes foot care centres are in operation in six regions covering 89% of the population (Table 2), and 7,567 people with diabetes were evaluated between 1 January 2010 and 31 March 2013. The project facilitated the introduction of HbA1c testing in the public system. Baseline data showed that 62.7% of participants had an HbA1c of <9% (75 mmol/mol), and 83.7% had a BP of <160/95. The number of major amputations was determined from operating room records for 42 months before the diabetic foot centre at GPHC opened in July 2008, when the mean monthly number of amputations was 7.95 (±standard derivation [SD] 4.05), and this fell significantly to 3.89 (±SD 2.30) in the 54 months after the DFC opened (data collected prospectively by GDFP from July 2008 until December 2012). After adjustment for an increased number of admissions, there was a 68% reduction in the average rate of major amputations in the first 22 months of the intervention compared to the preceding 30 months [20].

**Table 2:** GDFP database by region, female-to-male ratio (F:M), age, ethnicity and HbA1c.

	Region 1	Region 2*	Region 3*	Region 4*	Region 5*	Region 6*	Region 7	Region 8	Region 9	Region 10*	Total
**Population** ^†^ **(% total)**	26,941 (3.6)	46,810 (6.3)	107,416 (14.3)	313,429 (41.9)	49,723 (6.6)	109,431 (14.6)	20,280 (2.7)	10,190 (1.4)	24,941 (3.3)	39,452 (5.3)	748,613
**Number (region info)**	3	541	711	2,311	114	624	17	0	2	752	5,075**
**F:M**	1:2	2.41	2.27	1.93	2.35	1.93	0.88	NA	1.0	2.88	2.10^#^ (5,038:2,395)**
Age (SD) (range)**	Insufficient data	57.5 (12.0) (20.0–95.6)	55.4 (11.6) (18.2–80.7)	56.2 (12.4) (18.1–89.3)	51.3 (12.1) (18.3–80.4)	56.9 (11.1) (20.0–91.9)	Insufficient data	Insufficient data	Insufficient data	59.7 (11.9) (22.9–90.2)	55.7 (12.4) (18.1–99.7)
**Ethnicity (% total)**		456	569	622	66	577				664	2,954**
East Indian		335 (73.3)	423 (72.1)	272 (37.7)	32 (50)	455 (77.8)				68 (9.7)	1,585 (53.7%)
Afro-Guyanese		59 (12.9)	108 (18.4)	279 (38.7)	27 (42.2)	95 (16.2)				449 (64.1)	1,017 (34.3%)
Amerindian		8 (1.8)	2 (0.3)	16 (2.2)	2 (0.3)	2 (0.3)				62 (8.9)	92 (3.1%)
Mixed		26 (5.7)	34 (5.8)	53 (7.4)	5 (7.8)	24 (4.1)				83 (11.9)	225 (7.6%)
Other		28 (6.1)	2 (0.3)	2 (0.3)	0 (0)	1 (0.2)				2 (0.3)	35 (1.2%)**
Mean HbA1c -% SD (range)		9.8 (2.6) (5.1–16.2)	9.8 (3.1) (4.4–17.9)	8.6 (2.7) (4.3–19.2)	9.6 (3.0) (4.2–17.0)	8.7 (2.6) (4.7–15.2)				8.7 (2.5) (4.3–14.9)	8.6 (2.8) (4.1–19.2)

^#^Ostrow B, Sibbald RG, Taha N, Lowe J. Sex disaggregated diabetes prevalence study in Guyana needed. **Diabetes** Res Clin Pract 2014;106 (2):e17.

*regions with project intervention (DFC and trainings)

^†^2012 Census preliminary report available at http://www.statisticsguyana.gov.gy/census.html. Accessed 13 Dec 2014.

**Missing or error in data: Region 2,491; Gender 133; Age 2,284; and Ethnicity 4,612.

## Discussion—Successes and Challenges

### Project Model

This project demonstrates that K2A principles can make a major countrywide clinical impact by linking best clinical practices, QI, and implementation strategies. We identified one key component for success as being the need to translate new knowledge into changed behaviors inside the health care system. For example, after recognizing that limb salvage is a vital outcome, there was a marked, immediate, and sustained change in the behavior of surgeons faced with diabetic foot complications. A second key component is the commitment from the MoH to support diabetic foot centres and to embed the intervention components into the health care system through service agreements and training programs for doctors, medex, nurses, and rehabilitation assistants. This is a stellar example of government and health staff working together to create change. The third key component is the use of established training programs (IIWCC and Michener Institute courses) supplemented by on-site skills training, reflective practice, and continued mentorship in a staged support network to grow local expertise.

This project is unique in applying the K2A framework to a whole country. Social interaction allows all parties with a vested interest to be involved in adapting evidence to the local context, integrating research-based knowledge with experiential and contextual knowledge while maintaining the integrity of each. The K2A framework requires a systems perspective but does not presuppose linear flow, recognizing the complexity and multilayered processes that occur in health systems. Like real life, it can be challenging. In our project, facility renovation, education workshops, database development, and clinical team building as well as public engagement, to give but a few examples, progressed simultaneously but at different rates.

Challenges to improving diabetes care include promoting change in complex, entrenched individual and system activities. An IDF North America and Caribbean workshop in 2009 identified inadequate training and education, poor sharing of best practices between regions, difficulty in retaining specialists, inconsistencies in quality and delivery of care, and substandard government support and monitoring and evaluation as key challenges to improving diabetes care in the region [21]. We experienced all of these challenges. Barriers were overcome through workshops and consultations with project partners to identify problems with meeting project timelines and local partners’ priorities. We then worked collaboratively to overcome barriers based on these priorities.

While team building and education workshops progressed well, the major challenge faced was that for many reasons project partners were unable to meet timelines. Probably the biggest reason was the lack of an effective Ministry coordinator of CNCD during a large part of the project. This resulted in delays in completing foot centre renovations, implementing assessment tools, and resolving problems concerning data sharing. However, failure to understand Ministry procedures and the absence of such procedures in certain situations were other reasons. Another important problem was that trained health professionals migrated out of the country or moved to other MOH positions. The migration of KOLs out of the country was mitigated by the training of additional KOLs as well as the training of ten health care professionals in a diabetes educator course. While the project attempted to choose medex (physician assistants) rather than doctors, since the latter appeared to be the main émigrés, this was not always successful. As a result, we were continually confronted by human resource constraints with trained personnel being moved at short notice to other roles without consideration to their diabetes and foot care training status and importance to sustaining the new diabetes care system. Continuous negotiation at multiple levels was required, from clinic to the Minister of Health, and the best outcomes were achieved through face-to-face meetings. Project donor CIDA attended and facilitated important discussions. Data entry at two sites was delayed by theft of computer equipment; all entry points at all sites were secured after this. Other examples are given in the project reports to CIDA attached as supplementary information S1 Text (“Phase 1—Final narrative report”), S1 Table (“Table to confirm deliverables rendered—Phase 1”), S2 Text (“Phase 2—Final narrative report”).

One harm identified was a doubling of the rate of diabetic foot admissions at GPHC. The average monthly admissions rose from 21.2 before the DFC opened to 42 in the first 22 months of operation. Whether this represents the natural history of the diabetes pandemic in Guyana or increased access to the new treatment capacities is unknown.

## Plans for the Future

We would like to consolidate and further integrate previous innovations, expanding them to the 10% of the population living in hinterland regions. To this end, the pool of KOLs should be increased. We anticipate that as training reaches more health care professionals, thereby saturating the system, the movement of trained individuals will no longer matter.

While the specified interventions may be cost saving or cost-effective, this does not mean they are affordable for LMICs. We would therefore like to conduct cost analyses of our interventions. Under the leadership of the Banting and Best Diabetes Centre at the University of Toronto and the MoH Guyana, we have recently received a grant from the World Diabetes Foundation to expand our work to interventions described as cost saving or that cost less than US$1,500 per quality-adjusted life year (QALY), but which pose some feasibility challenges [5]. This will include developing local capacity to screen, diagnose, and treat diabetic retinopathy and provide antepartum care for women with diabetes, screening for gestational diabetes, and lifestyle interventions to prevent Type 2 diabetes. To this end we have developed new partnerships with the international eye care nongovernmental organization (NGO) Orbis International and the the Women, Neonates, Diversity, Outreach, Opportunities, and Research (WONDOOR) Global Health program of the University Hospitals, Cleveland, United States, which is facilitating an obstetrics and gynecology postgraduate training program at GPHC. Finally, we would test the transferability of our work in another LMIC with a larger population and a different health system.

The challenge of diabetes in LMIC requires rapid deployment of proven interventions that are cost saving or cost-effective. This requires empirical research in a variety of contexts. Our program contributes to an existing body of work on foot care including the IDF Step by Step program (http://www.idf.org/webdata/docs/Step_by_Step_article.pdf) and several projects supported by the World Diabetes Foundation (Table 3).

**Table 3:** Comparison of Foot Care Projects.

	Step by Step^1^	Guyana Diabetes and Foot Care Project	The Samadhan System^2^
**Brief Description**	Incorporates longitudinal training of HCP with follow-up sessions and hands-on practical training.	Similar educational approach to Step by Step using a formal key opinion leader team (“train the trainer model”). Builds capacity in the public-funded health system through facility development and provision of new clinical tools.	Encourages one specialist to gain knowledge in all areas of diabetes care, including nutritional and podiatric education, in order to provide adequate care in a resource-limited setting.
	Encourages provider and patient education for knowledge dissemination	Integrated into Ministry of Health Strategic Plan.	
**Location**	Tanzania, India, Sri Lanka, Nepal, and Bangladesh	Guyana	India
**Sustainability and Capacity Building**	Yes	Yes	No
**Multidisciplinary**	Yes	Yes	No
**Education**	Yes	Yes	No
**Main Differences**	• Restricted to diabetic foot management	• Integrates diabetic foot care into CNCD care in public-funded health system	A cheap (US$1), effective, and simple to use offloading tool was designed and used by author
	• Spans multiple countries	• Facilitates capacity building through infrastructure (clinic) development and introduction of new clinical tools (e.g., HbA1c testing and plantar pressure redistribution devices)	

^1^Abbas ZG, Lutale JK, Bakker K, Baker N, Archibald LK. The “Step by Step” Diabetic Foot project in Tanzania: a model for improving patient outcomes in less developed countries. Int Wound J. 2011;8:169–175

^2^ Shankhdhar K. Improvisation is the key to success: The Samadhan system. Adv Skin Wound Care. 2006;19(7): 379–383

Using a unique application of quality improvement methods on a nationwide scale, we demonstrated that it is possible to introduce best practice methods and achieve sustained improvements in care for foot ulcers. Marked and sustained reduction both in major amputation numbers and in the proportion of inpatients with diabetic foot complications requiring major amputation verifies the improvement. Improvements in diabetes and hypertension control will take longer to establish.

## Supporting Information

S1 Table Table to confirm deliverables rendered—Phase 1. (DOC)

S1 Text Phase 1—Final narrative report. (DOC)

S2 Text Phase 2—Final narrative report. (DOC)

## Abbreviations

BP: blood pressure
CCM: chronic care model
CEO: chief executive officer
CIDA: Canadian International Development Agency
CNCD: chronic noncommunicable diseases
CME: continuous medical education
DFC: diabetic foot centre
F:M: female-to-male ratio
GDFP: Guyana Diabetes and Foot Care Project
GPHC: Georgetown Public Hospital Corporation
HbA1c: glycosylated hemoglobin
HCP: health care professional
IDF: International Diabetes Federation
IIWCC: International Interprofessional Wound Care Course
K2A: knowledge to action
KOL: key opinion leader
LEA: lower extremity amputation
LMIC: low- and middle-income countries
MoH: Ministry of Health
NGO: nongovernmental organization
PAHO: Pan American Health Organization
PPR: plantar pressure redistribution
PWD: person with diabetes
QALY: quality-adjusted life year
QI: quality improvement
SD: standard deviation
VIP: vascular, infection/inflammation, pressure
WONDOOR: Women, Neonates, Diversity, Outreach, Opportunities and Research
