# Supplementary material for: The Guyana Diabetes and Foot Care Project: A Complex Quality Improvement Intervention to Decrease Diabetes-Related Major Lower Extremity Amputations and Improve Diabetes Care in a Lower-Middle-Income Country
Source: PLoS Med. 2015 Apr 21;12(4):e1001814. doi: 10.1371/journal.pmed.1001814 (PMC4405371; doi:10.1371/journal.pmed.1001814)
Supplement: S1 Table — (DOC) [file pmed.1001814.s001.doc]

| ***TASKS*** | ***EXPECTED***  ***DELIVERABLES*** | ***ACTUAL OUTPUTS AND DELIVERABLES*** | ***AUTHORITY FROM Ministry of Finance***  ***I certify that services and deliverables were rendered with satisfaction*** |
| --- | --- | --- | --- |
|  | |  |  |
| ***Activity 1: Prerequisite Conditions - Functioning DFC/Project Office*** | - Rent to own office equipment - Hire and train secretary - Financial accounting - Outfitted foot centre - HbA1c testing GPHC - Footwear source | - *Equipment rented* - *Secretary hired, trained – now on 2^nd^ secretary* - *All financial documents submitted* - *Centre outfitted* - *Testing capacity acquired March 2010* - *Procurement of offloading supplies functional* | - *Yes* □ *% completed:* - *No*  □   *If no, please explain why.* |
| ***Activity 2: Screening in Medical Diabetic Outpatient Clinic*** | - Regular screening of patients in medical diabetes clinic using 60 second tool - Screening too created - Screening spreadsheet created | - *1226 patients screened(Feb 2010) but sustainable ongoing screening in medical clinic staff not achieved – shortage of staff, disruptions by construction – attempts ongoing* - *Tool functional* - *Spreadsheet functional* | - *Yes* □ *% completed:* - *No*  □   *If not, please explain why.* |
| ***Activity 3: DFC Leadership and Interprofessional Diabetic foot teams*** | - 1 cohorts (4) key opinion leaders identified and trained - DFC staffed with interprofessional teams | - *2 cohorts (8) key opinion leaders identified and trained* - *DFC staffed* | - *Yes* □ *% completed:* - *No*  □   *If no, please explain why.* |
| ***Activity 4.1: Training visits by Canadian specialists*** | - 3 training visits by Canadian wound care specialists | - ***4*** *training visits by Canadian wound care specialists* | - *Yes* □ *% completed:* - *No*  □   *If no, please explain why.* |
| ***Activity 4.2: International Interprofessional Wound Care Course (IIWCC)*** | - 1 cohort complete training | - *2 cohorts completed training: 1^st^ cohort 75% certificate of completion ( 1 to complete selective); 2^nd^ cohort: in process – completion date Dec. 2010* | - *Yes* □ *% completed:* - *No*  □   *If no, please explain why.* |
| ***Activity 5: Operation Diabetic Foot Centre*** | - *Functional DFC seeing patients* | - *1516 patients assessed and treated by April 30, 2010* - *6075 patient visits since July 2008* | - *Yes* □ *% completed:* - *No*  □   *If no, please explain why.* |
| ***Activity 6: Assessment Tools*** | - Tools created | - *5 tools created* | - *Yes* □ *% completed:* - *No*  □   *If no, please explain why.* |
| ***Activity 7: Amalgamation with National Diabetes Program*** | - Ministry pamphlets edited | - *Ministry pamphlets edited* - *60 second tool used nationally* - *Phase 2 regionalization Project commenced March 2010* | - *Yes* □ *% completed:* - *No*  □   *If no, please explain why.* |
| ***Activity 8: Continuing education in Guyana*** | - 2 workshops | - *1 workshop held* - *5 CME presentations made* | - *Yes* □ *% completed:* - *No*  □   *If no, please explain why.* |
| ***Activity 9: Post-project analysis and dissemination*** | - Not specified | - *2 journal articles produced* - *10 presentations made at 7 conferences* | - *Yes* □ *% completed:* - *No*  □   *If no, please explain why.* |
|  |  |  |  |
|  | |  | - *Yes* □ *% completed:* - *No*  □   *If no, please explain why.* |
|  |  |  |  |

_________________________

Signature

_________________________

Date
