# Supplementary material for: The Guyana Diabetes and Foot Care Project: A Complex Quality Improvement Intervention to Decrease Diabetes-Related Major Lower Extremity Amputations and Improve Diabetes Care in a Lower-Middle-Income Country
Source: PLoS Med. 2015 Apr 21;12(4):e1001814. doi: 10.1371/journal.pmed.1001814 (PMC4405371; doi:10.1371/journal.pmed.1001814)
Supplement: S1 Text — (DOC) [file pmed.1001814.s002.doc]

**CCF Project No 530-0272-G015**

**Technical Assistance for the Development of an enhanced diabetic foot program at GPHC in Guyana**

**Final Narrative Report**

**Guyana Diabetic Foot Project – Phase 1**

**February 15, 2008 – May 31, 2010**

**The Guyana Diabetic Foot Project Team**

| **Canadian team** | |
| --- | --- |
| **PWGSC** | - **Philippe Kanonko**, **International Services, Government Consulting Services** |
| **CCF** | - **Daniel Wallace, project officer** |
| **MDs/ Project Leaders** | - **Brian Ostrow, Office of International Surgery, University of Toronto** - **R. Gary Sibbald, Professor, Dalla Lana School of Public Health,**   **University of Toronto** |
| **Nursing** | - **Kevin Woo, PhD, clinical scientist, Dalla Lana School of Public Health, University of Toronto** - **Marjorie Fierheller – wound care specialist, Humber River Regional Hospital** - **Pat Coutts, Mississauga clinic, President elect CAWC** |
| **Rehab &**  **Supporting Staff** | - **Laura Lee Kozody – chiropodist, Trillium Health Center, Mississauga** - **Other Rehab, epidemiologists + + +** |
| **Guyana Team**  ***Key opinion leader** | |
| **MOH** | - **Dr. Leslie Ramsammy, Minister of Health** - **Dr. Gumti Krishendat – Chronic Diseases Unit, Ministry of Health** |
| **GPHC** | - **Mr. Michael Khan, CEO** - **Dr. Madan Rambaran – Director, Medical and Professional Services** |
| **MDs** | - **Carlos Martin* - physician lead, Diabetic Foot Centre (DFC)** - **Chris Persaud* - junior surgeon** |
| **Nurses** | - **Melinda Thomas* - nurse, DFC** - **Alexis Reid *- nurse, DFC** - **Sabrina Brummell* - nurse, Enmore Polyclinic** |
| **Rehab,**  **Other** | - **Debita Harripersaud* - physiotherapist, DFC** - **Amanda LaRose* - rehab assistant** - **Jacqueline Williams* - diabetes educator, Ministry of Health** - **Shabana Shaw – secretary, DFC** - **Nerica Bassodeo, Director, Medical Information, GPHC** - **Tallim Karimullah, Director, Finance department, GPHC** |

The authors wish to thank all those who contributed to the success of the Guyana Diabetic Foot Project and dedicate this Report to their efforts.

**Table of Contents**

1. **Introduction**
2. **Background and Objectives**
   1. **Background and Rationale**
   2. **Goal and Purpose**
   3. **Results**

**2.3.1. Project Goal**

**2.3.2. Developmental outcomes**

1. **Summary Description of the Project**
   1. **Management and Organization**
   2. **Activities**
      1. **Activity 1: Prerequisite Conditions – Establishing a functional Diabetic Foot Center (DFC)/Project Office**

**Activity 1.1: Infrastructure**

**Activity 1.2: Training of secretary**

**Activity 1.3: Financial accounting & templates**

**Activity 1.4: Outfitted DFC**

**Activity 1.5: HbA1C testing**

**Activity 1.6: Footwear source**

- - 1. **Activity 2: Screening in Medical Diabetic Outpatient Clinic**
    2. **Activity 3: DFC Leadership and Inter-professional Diabetic foot teams**
    3. **Activity 4.1: Training visits by Canadian specialists**
    4. **Activity 4.2: International Interprofessional Wound Care Course (IIWCC)**
    5. **Activity 5: Operation Diabetic Foot Centre**
    6. **Activity 6: Diabetic Foot Assessment Tools**
    7. **Activity 7: Amalgamation with National Diabetes Program**
    8. **Activity 8: Continuing education in Guyana**
    9. **Activity 9: Post-project analysis & Dissemination**
  1. **Resources utilized and responsibilities**
     1. **Subcontractors**
        1. **Hiring process**

**3.3.2. Stakeholders**

1. **Analytical Review of the Project**
   1. **Project Rationale and justification**
   2. **Results**
      1. **Expected versus Actual Outcomes**

**Table of contents (continued)**

**4.2.3. Expertise Utilized**

- 1. **Intellectual property**
  2. **Political considerations**
  3. **Gender Equality issues and results**
  4. **Disbursements**

**4.6.1. In-kind contributions**

- 1. **Scheduling and logistical difficulties**
  2. **Knowledge sharing results**
  3. **Synergy**

**5. Conclusions**

**Abbreviations:**

CA – Contribution Agreement

CAGS – Canadian Association of General Surgeons

CAWC – Canadian Association of Wound Care

CCF –Caribbean-Canadian Cooperation Fund

CE- Continuing Education

CIDA – Canadian International Development Agency

CME – Continuing Medical Education

CPE- Continuing Professional Education

DFC – Diabetic Foot Center

DFU – Diabetic foot ulcer

GPHC – Georgetown Public Hospital Corporation

IIWCC – International Inter-professional Wound Care Course

KOLs – key opinion leader

MOH – Ministry of Health

NCD – Non-Communicable Diseases

PoA – Plan of Action

PPR – Plantar Pressure Redistribution

PWGSC – Public Works Government Services Canada

VOIP – Voice over Internet Protocol

WCH – Women’s College Hospital

**1. Introduction**

CCF Project G015, the “Guyana Diabetic Foot Project”, was designed to enhance diabetic foot care patient outcomes at the Georgetown Public Hospital in Guyana, South America. The project was conducted from February 15, 2008 to May 31, 2010 and was funded by the Caribbean-Canadian Cooperation Fund, a branch of Canadian International Development Agency (CIDA). As will become evident from this Report, the Project had a very significant impact on diabetic foot care patient outcomes and health care professional performance in Guyana. The completed program has become Phase 1 of a larger Phase 2 project, funded by the Partnership Branch of CIDA, to regionalize the Interprofessional team approach to community-based diabetes care throughout Guyana,

The Final Narrative Report is the appropriate documentation to sum up the achievements of this Project. It is an opportunity to reflect on the actual results in relation to the initial Goal and Objectives. Reflection will be the means to evaluate and to critique all aspects of the Project in order to determine, not only how the Goal and Objectives could have been optimized, but also whether they were the appropriate Goal and Objectives. The Project documents - the Contribution Agreement (CA), its Amendments (Amend#1&2), the Plan of Action (PoA) and the 9 subcontractors’ quarterly reports - form the source material for this Report and are all readily available. The opinions expressed are those of the authors.

**2. Background and Objectives**

**2.1. Background and Rationale**

The Guyana diabetic foot project started as the vision of Dr. Madan Rambaran, Director of Medical and Professional Services at Georgetown Public Hospital Corporation (GPHC). He interested retired Canadian surgeon, Dr. Brian Ostrow, who became the project co-ordinator and champion. Dr. Ostrow visited Guyana in January 2007, as one of the Canadian mentors for the surgical diploma course (also supported by CCF); he carried out a needs assessment and submitted the successful proposal to the Caribbean Canadian Cooperation Fund (CCF). He recruited Dr. Gary Sibbald, a Canadian physician and a world-renowned wound care expert, to become the project team leader.

In 2010, there is recognition that type II diabetes represents a great threat to the health and livelihood of the world’s people, especially those in the low and middle income countries. In 2008, the General Assembly of the United Nations developed an Action Plan for the Prevention and Management of Non-Communicable Diseases (NCD). (1) This was not the case in 2006-7 when the Project was conceived. At that time the development community, including CIDA, was more concerned with communicable diseases rather than chronic diseases as a health priority in developing countries. The negative effect that NCD had on the Millennium Development Goals was just beginning to be appreciated.

Despite inadequate statistics, the heavy burden of diabetes in the Caribbean is well- known. The Caribbean region has some of the highest rates of diabetes-related major lower limb amputations in the world. (2) In Guyana, at the GPHC, diabetic foot complications represented the single largest reason for admission (10%) to a surgical ward. At any one time, 30% of surgical beds were occupied by these patients. (3) Forty-two percent of these patients went on to some form of amputation; half of which were major amputations. (4)

The Best Practice Recommendations for the Prevention, Diagnosis and Treatment of Diabetic Foot Ulcers, published by the Canadian Association of Wound Care (CAWC) were chosen as the theoretical basis of the Project. These recommendations are based on evidence informed enablers for practice derived from the evidence based guidelines of the Registered Nurses Association of Ontario. The methodological quality of these guidelines has been validated with the Agree Tool. ([www.agreecollaboration.org](http://www.agreecollaboration.org/) ) In the developed world, the creation of multidisciplinary diabetic clinics resources has been shown to decrease amputation rates in persons with diabetes by over 50%.(5) However there is a paucity of information concerning the effect of these clinics in low and middle income countries. (6)

**2.2. Goal and Purpose**

The Goal (ultimate outcome) of the Project was expressed on page 6 of the CA,

*“****The project aims to improve*** ***the overall health of persons with diabetes in Guyana by developing an enhanced diabetic foot program at the Georgetown Public Hospital Corporation (GPHC)****.”*

The Purposes (objectives or intermediate outcomes) of the Project were also defined on page 6-7 of the CA:

***1. Create effective outpatient GPHC capacity, which uses the Best Practices, in the risk assessment, early diagnosis and education of people with diabetes, to prevent foot ulcers***

***2. Create inpatient/outpatient GPHC capacity, which uses the Best Practices, to effectively assess and treat diabetic foot ulcers***

***3. Create capacity in GPHC and Ptolemy-Reid Rehabilitation Centre to effectively reduce plantar foot pressures in all grades of DFU in order to facilitate healing***

***4. Educate the Guyanese medical community about the scientific basis of the Best Practices and all facets of the management of DFU***

Subsequent to the signing of the CA and the appointment of the two volunteer subcontractors, a Plan of Action (PoA) was developed. This PoA put forward the following principles for the Project (p.6, PoA):

1. **Creation of inter-professional diabetic foot teams comprised of doctor, nurse and rehabilitation foot specialist.**
2. **Improve and integrate current resources for diabetic foot care.**
3. **Amalgamation with the National Program for Control and Management of Diabetes in Guyana.**
4. **Integrate proven educational strategies based on general training of healthcare professionals and the identification of key opinion leaders.**
5. **Introduction of the new capacity in a sustainable manner.**

On the basis of these principles, the PoA confirmed the original Project objectives, but substantially altered the original activities, their immediate outcomes and the performance indicators associated with them, as had been set out in the CA. These were changed to reflect the importance of the newly articulated Project principles, especially the interprofessional team and key opinion leader (KOL) models, as well as context specificity, sustainability and amalgamation with the Ministry of Health’s (MoH) national diabetes program. These adjustments combine the Diabetic Foot scientific literature with the evidence-based educational process literature, (as defined in the Cochrane reviews of educational methodology), that have been shown to improve healthcare professional performance and patient outcomes.

The Project objectives are now situated inside the development of a Diabetic Foot Centre (DFC). The DFC becomes the sustainable entity with the capacity to deliver Project objectives on an ongoing basis. To achieve these objectives the PoA outlined in detail nine activities which form the basis of the Project and are described below in **3.2.** The PoA was adopted by GPHC and approved by CCF resulting in Amendment #1 of the CA. The PoA had considerable financial implications which are dealt with in **4.7.** On the recommendation of the Minister of Health it was decided that, if the DFC could objectively be shown to have this capacity by the end of the Project, it would be declared a **Centre of Excellence in Diabetic Foot Care.** All further analysis of Project results in this Report will be based on the outputs and developmental outcomes as defined in **Articles 12.1 &12.2** of Amendment #1.

**2.3. Results**

The measurement of project results - outputs, immediate, intermediate and, where possible, ultimate outcomes – are based on performance indicators. Completed activities (outputs, deliverables) rather than developmental outcomes are specified in **12.1** (Amend#1). The outputs of completed activities are best discussed with these activities and this will be outlined in **3.2.**below.

In **12.2** (Amend#1) the various levels of developmental outcomes have been condensed into a single entity. Moreover, sadly, not all designated indicators were operationalized into the Project. Therefore it is impossible to present information on: the number of patients with foot ulcers (DFU) presenting and/or being admitted to GPHC, their length of stay, the healing rates of ulcers and the foot status classification of diabetes patients referred to GPHC. An analysis of the causes for this weakness is presented in **4.2.** That being said, a significant amount of information is available to describe Project developmental results on various levels. The approach taken here is a descriptive one with a discussion of project goal and developmental outcomes.

**2.3.1. Project goal –** the main objective indicator available, that shows ***“the overall health of persons with diabetes in Guyana”*** has been improved by the Project, is the major amputation rate at GPHC. Amputations of all kinds are strong indicators of negative health outcome and major amputations; those above and below the knee, are especially indicative of negative outcomes. The Project was able to accurately measure the monthly rates of diabetes-related major amputation at the GPHC since 2005. This analysis demonstrated that, before the DFC opened, the mean monthly average was 7.95 amputations per month. The post DFC monthly average, for the 17 project months up to December 2009, fell to 4.29. This represents a 46% decrease and is highly significant (p=.0001). The details of this analysis are shown in **Table 1** and **Figure 1.** The data did not demonstrate a seasonal variation. While various assumptions must be made to claim causality, these results strongly indicate Project effect with an estimated average of 3.66 limbs saved each month since the start of the Project.

**Table 1 Comparison of mean monthly major amputation rates before and after DFC**

| Risk | Months | Mean | Est. St dev | 95% CI | Range of true population mean | | T test |
| --- | --- | --- | --- | --- | --- | --- | --- |
| Before DFC | 43 | 7.953488 | 3.9997231 | 1.19 | 6.75 | 9.15 | P =  0.000144 |
| After DFC | 17 | 4.294118 | 2.6164075 | 1.2 | 3.05 | 5.53 |

**Figure 1**

**DM-related Major Amp GPHC 2005-end2009**

0

1

2

3

4

5

6

7

8

9

10

**Before DFC After DFC**

**Mean # persons-amp/month**

Before DFC

After DFC

This reduction in major amputations at GPHC is made more significant considering the ongoing increase in admissions to the hospital for diabetic foot complications. **Table 2** shows that, since 2006, annual admission to GPHC for foot complications has increased by 70%. This increase predated the Project and is a reflection of the high and increasing burden of foot disease in Guyana. The admission criteria did not change, patients are admitted for limb threatening foot complications, and the DFC is not the primary source of admissions. Against this increasing burden, the Project was able to decrease the number of major amputations by 46%. A reduction in DFU admissions in the future would be a sign of effective prevention activities.

**Table 2**

| **Annual Diabetic Foot Ulcer Admissions to GPHC** | | | | |
| --- | --- | --- | --- | --- |
| **Year** | 2006 | 2007 | 2008 | 2009 |
| **# admissions** | 203 | 259 | 272 | 347 |

**2.3.2. Developmental outcomes:**

**a) Effective, outpatient GPHC capacity by implementing an Interprofessional Best Practices program for risk assessment, early diagnosis and education of people with diabetes to prevent foot ulcers**

The main indicators of the capacity of the Project to prevent DFU relate to screening and risk assessment. A unique context specific 60 second screening tool was developed by the project team leader. Screening in the diabetic outpatient clinic at GPHC, using the 60 second tool, began in April 2008. By March 2010, 1226 patients had been screened. The clinic has a population base of 2000 patients. The profiles of the first 1000 screened patients are tabulated in **Tables 3-5.** The mean age was 57.5, a decade younger than some North American reported patient series. Seventy-one percent of screened patients were women and only 29% are men. Data from the Diabetic Atlas shows that the estimated gender ratios for type II diabetes in Guyana are: 54% female; 46% male.(7) This indicates that men do not present for screening. From the profiles in **Table 5**, it is important to note that 7.7% of screened patients have diabetic foot ulcers that were previously undetected by their healthcare providers and often the patients were not aware of their presence. A cumulative 47% of patients are considered high risk and 66% of these have received an appointment to the DFC for assessment and treatment. Patient education using both verbal and written strategies is standard practice at the DFC.

**Table 3 Gender ratios Table 4**


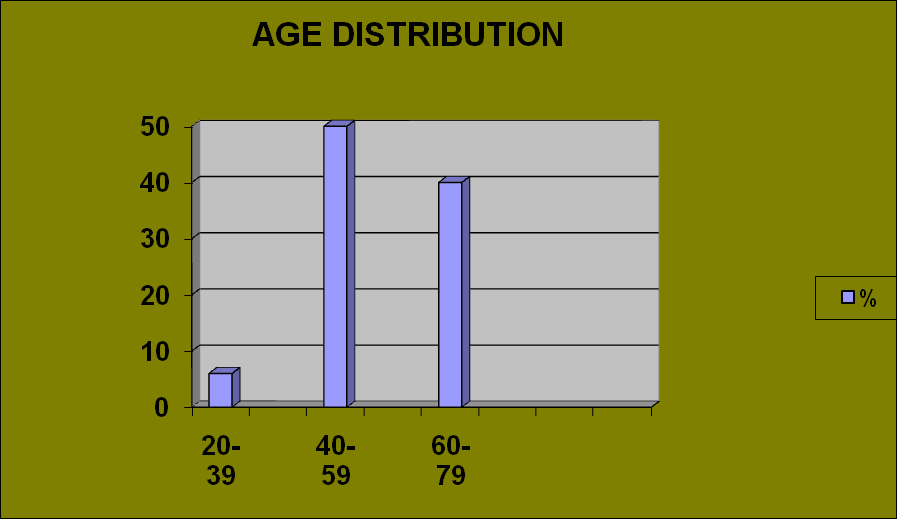


| **Gender** | **%Female** | **% Male** |
| --- | --- | --- |
| **Total screened** | 71 | 29 |
| **Low-risk** | 73 | 27 |
| **High risk** | 67 | 33 |

**Table 5 Risk Profile of the first 1000**

**screened patients**

| **ITEM** | **NO %** | **YES %** |
| --- | --- | --- |
| **Previous Ulcer** | 86.5 | 13.5 |
| **Previous Amp** | 95 | 5 |
| **Absent pulse** | 91.5 | 8.5 |
| **Stiffness ankle/toe** | 98.7 | 1.3 |
| **Active DFU** | 92.3 | **7.7** |
| **Ingrown toenail** | 81.7 | 18.3 |
| **Callus** | 77.7 | 22.3 |
| **Fissure** | 89.5 | 10.5 |
| **Neuropathy** | 76.6 | 23.4 |
| **REFERRED DFC** | **59.1** | **40.9** |

**b) Effective, inpatient and outpatient, GPHC capacity which uses the Best Practices to assess and to treat diabetic foot ulcers (DFU)**

The available indicators to assess this outcome are those which reflect the function of the DFC, its hours, staffing, the numbers and types of patients seen and those which show a Project effect in improving clinical practice towards inpatients with DFU. It should be pointed out that patients admitted to GPHC with DFU have, in general, very deep advanced ulcers with deep tissue infection and often osteomyelitis or infection of the bone. (Grade 2-3, Stage C or D by University of Texas classification).

The Diabetic Foot Center (DFC) is a 4 room outpatient facility in the GPHC, fashioned out of a two rehabilitated minor operating rooms. It is composed of a registration room and 3 clinical rooms – two of which are used for assessment and treatment of ulcer patients and the third for assessment of plantar pressure redistribution needs of the patients as well as storage of wound care and plantar pressure redistribution supplies. **See photos 1-4.**


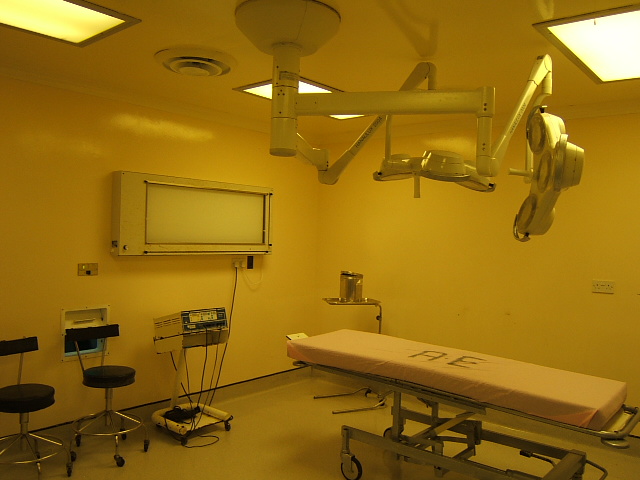


**Photo 1 Clinical room before rehab**


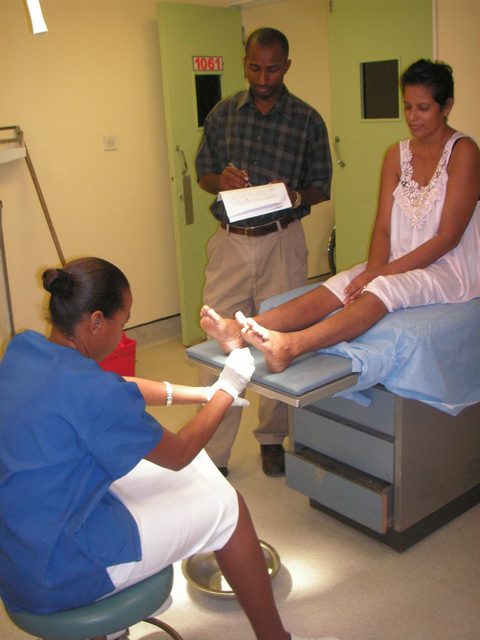


**Photo 2 Clinical room in use**


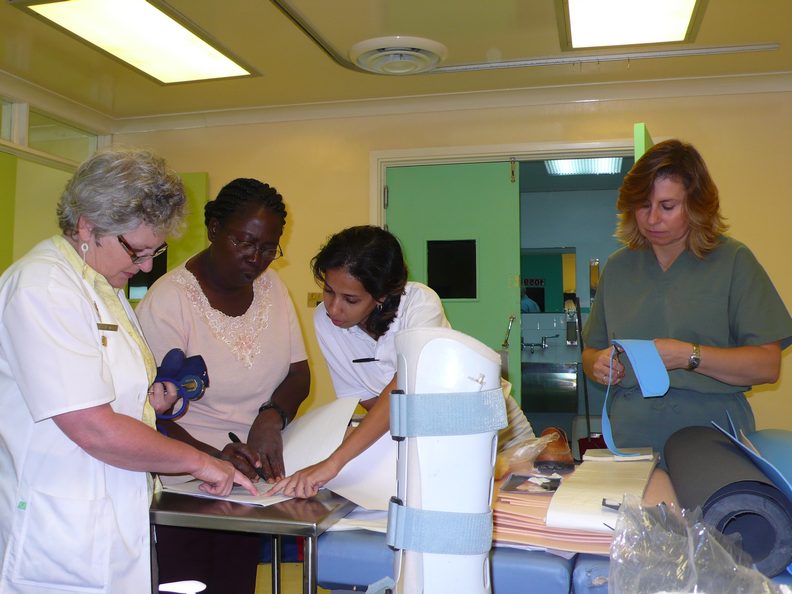


**Photo 3 Plantar pressure redistribution material in use**


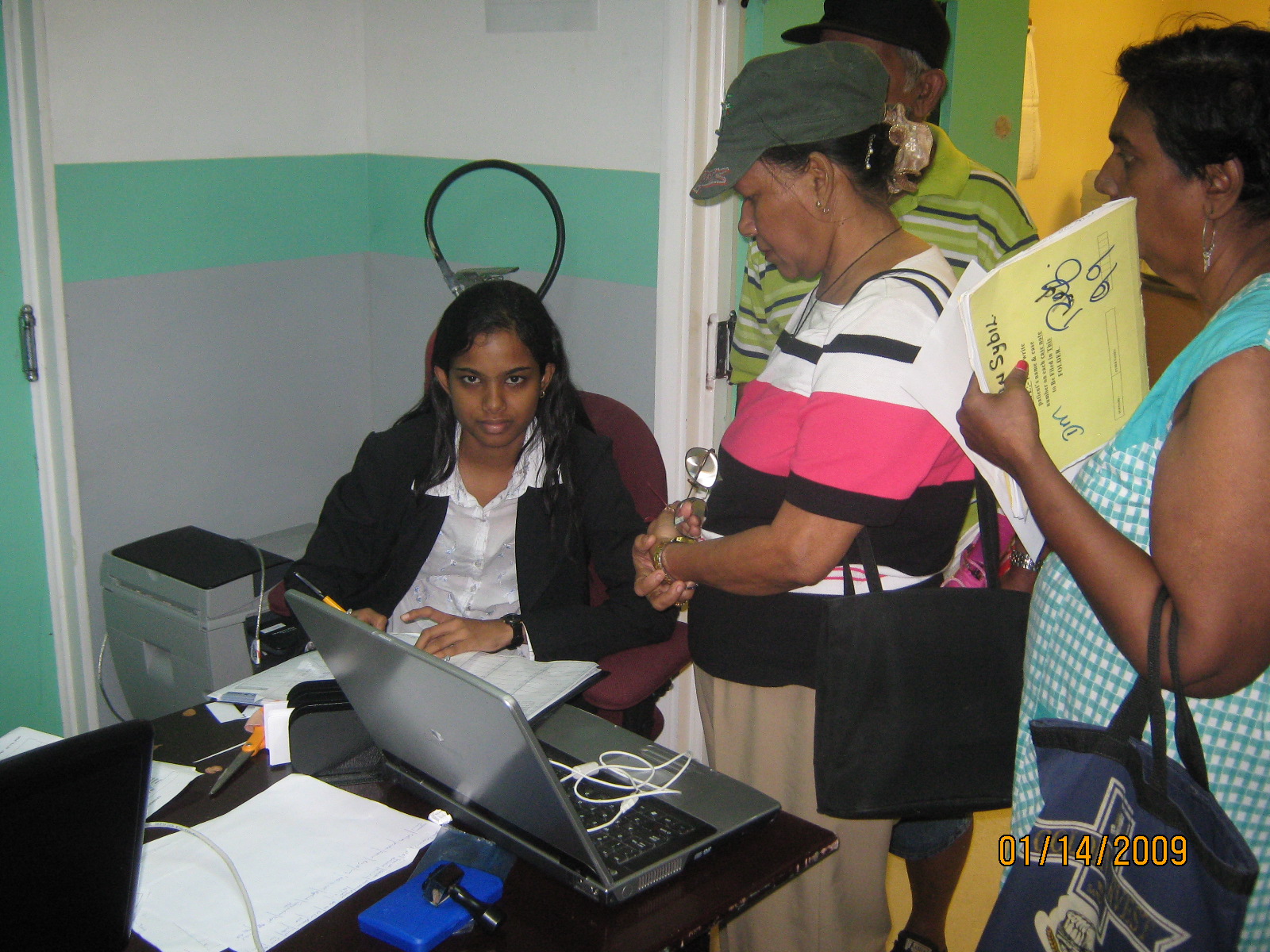


**Photo 4 DFC office in operation**

The DFC operates 5 days a week from 8:30am to 4:30pm. It is staffed by a secretary and permanent members of the key opinion leader team comprised of a physician, nurse, physiotherapist and diabetic educator employed primarily by the Ministry of Health (F/M=3). To adequately staff the clinic, a total of 50 health professionals were trained during the Project to work part time at the DFC and to care for the inpatients. The professional breakdown is 15 doctors; 25 nurses; 9 rehabilitation related specialists; 1 nurse educator (F/M=1.5). The DFC is staffed with about 65 person shifts/week (1 shift = 4 hours AM or PM), or about 6 persons/shift with the reasonably successful objective of having all Interprofessional team members present for each shift. The overall absenteeism rate ranges from 15-20% per quarter with the rehabilitation component being the most difficult to staff due to the competing priorities and paucity of personnel in this sector.

The DFC opened on July 21, 2008 simultaneously with the 1^st^ training visit. The clinic has assessed and treated a total of 1516 discrete patients as of April 30, 2010. Of these 61% were female. The DFC treated a total of 285 inpatients during this period or 19% of the Centre’s total population. The percentage of men in this inpatient group is 53%, whereas the percentage of men in the screened group is only 29%. This indicates that men do not come for screening; that they have an excessive risk of major ulceration or wait longer with more advanced disease to come for treatment. Probably all of these assumptions are correct. Of the total patient population 18% were high risk patients without ulcers, who had been screened in the medical outpatient clinic. This leaves about 60% of patients coming from outside of the GPHC system. This reflects the heavy burden of foot disease in the community and the need for the Phase 2 of the Project.

As of March 22, 2010 there were a cumulative 6075 patient-visits to the DFC or an average of 13.6 visits per day. The average number of new patients seen per week varied between 12 and 16. All these figures reflect the extremely active clinical role of the DFC.

Care plans for inpatients with DFU, are created in consultation between DFC and the surgical staff. They are based on the adapted Best Practices and use the enablers and assessment forms created by the Project. Surgical debridement restricted to the foot takes place in the DFC as does regular follow-up for in hospital patients and for these individuals after discharge. All 11 surgical residents, as well as 12 inpatient surgery ward nurses, received training by the Project. While weaknesses still exist, there is an overall consensus that the care of inpatients has become more co-ordinated, uniform and appropriate. A study, conducted as a selective for the International Interprofessional Wound Care Course (IIWCC) by one of the 1^st^ cohort KOLs, demonstrated improved appropriateness of antibiotics prescription after training. (Personal communication)

**c) Capacity in GPHC and Ptolemy-Reid Rehabilitation Centre to effectively reduce plantar foot pressures in all grades of DFU in order to facilitate healing**

There are three main components in the assessment and treatment of Diabetic Foot Ulcers known as the VIPs (Vascular Supply, Infection, Plantar Pressure Redistribution and sharp surgical debridement). The introduction of plantar pressure redistribution (PPR) for patients with DFU was extremely important because this is a vital therapeutic modality in diabetic foot care that was previously unknown in Guyana. It was a challenge because of a complete absence of professional foot specialists in Guyana, chiropodists or podiatrists, as well as a shortage of professional physiotherapists. The project trained a professional physiotherapist, a mixture of prosthetic technicians from the Ptolemy-Reid Rehab Unit, cast room technicians and rehab assistants (9 in all, F/M=4/5).

The PPR standard of care for DFU in North America is a total contact cast or pneumatic walker that may be made irremovable (diabetic walker). The later was too expensive and the former required too much expertise for the training period, is contraindicated with deep infection or peripheral vascular disease, eliminating a large number of the patients in the DFC.

For the Guyana project, we needed materials that were relatively inexpensive and easy for patients to be adherent to their use in a warm and often humid tropical climate. Therefore offloading sandals specifically designed for forefoot and hind foot ulcers were made available for clinic patients. The Interprofessional teams in general and the rehab specialists in particular were trained in the use of these. The rehab specialty team acquired skills to make custom orthotic insoles as well. These pressure redistribution devices were cost-effective and economically sustainable in the Guyana context.

The supply of these devices was not part of the Project as such. The Minister of Health made the commitment to provide these sandals free of charge to patients for the first year of the Project. Logistical procurement was undertaken by the subcontractors with the declared intention to hand over procurement to the Ministry of Health as of November 2009. During the course of the 21 months (end of April 2010) of DFC operation a total of 420 sandals were prescribed or about 20/month. While no new sandals have been purchased as yet by the GPHC (65 remaining as of April 2010) there are signs that this system is being put into place for the Phase 2 Project.

**d) Capacity to educate the Guyanese medical community, about the scientific basis and current expert knowledge of the Best Practices, and all facets of the management of DFU**

While Amendment #1 predicted that two planned Continuing Medical Education (CME) workshops would result in Best Practices being implemented throughout Guyana with earlier rates of referral, specific data on this indicator is not readily available. The 60% of DFC patients that came from outside the GPHC system are an indication of the Centre’s expertise and recognition by the Guyana medical community. A total of five CME events on the management of the diabetic foot were held during the Project and will be discussed in **3.2.9.**

Looking towards the Phase 2 proposal to regionalize diabetic foot care throughout Guyana, submitted in June 2009, an interactive lecture, workshop and clinical program for health professionals outside GPHC took place in November 2009. Under the supervision of Project team leader, Dr. Gary Sibbald, the 1^st^ cohort of KOLs undertook a 3 day workshop on the diabetic foot for 15 health professionals (4 doctors, 7 nurses and 4 rehab specialists; F/M=12/3). The Guyanese KOL team were the primary educators for this interactive lecture, workshop and clinical program. It was extremely successful. A pre- and post- workshop test was carried out to assess knowledge gained. The mean pre-test score was 41%; the mean post test was 66%.

The training activities of the KOL team have not been limited to those organized within the Project. Formal training sessions have been organized for medical students at the University of Guyana and offshore. An organized rotation of nursing students, medical interns and interns through the DFC takes place. Plans also exist for training of medex students. Medex are doctor equivalents in Guyana and are nurses with advanced training. In the opinion of the subcontractors these activities confirm the capacity of the DFC and the KOL team to educate the Guyanese medical community.

**e) Centre of Excellence in Diabetic Foot Care**

During November 2009 Dr. Sibbald carried out a review of DFC to see if it could be designated as a Centre of Excellence. The following criteria were considered significant:

1. Comprehensive diabetic foot ulcer screening
2. Patient-centered diabetic foot ulcer treatment program with an inter-professional model
3. Care plans based on applying Best Practices adapted to local conditions
4. Local capacity to train new health care professionals in diabetic foot ulcer prevention, assessment, diagnosis, treatment and follow up.
5. Provision of context specific and affordable plantar pressure redistribution toolkit.
6. HbA1c testing to monitor long term diabetic control
7. Adequate data collection systems

Because HbA1c testing was not available in November 2009 only a provisional designation was possible. Since HbA1c testing began in April 2010, the review will be revisited in July 2010.

**3. Summary Description of the Project**

**3.1. Management and Organization**

Under the rules of the Caribbean Canadian Cooperation Fund (CCF), the Guyana organization was the lead organization. Therefore under the terms the Contribution Agreement (CA), the Georgetown Public Hospital Corporation was designated the lead organization and Dr. Madan Rambaran, the project manger. Two volunteer subcontractors were chosen: Dr. Brian Ostrow as project coordinator and Dr. Gary Sibbald as project team leader. These subcontractors in collaboration with GPHC management and other Guyanese partners developed the Plan of Action, organized all project activities and submitted the nine quarterly Project reports, on time and as required. The Finance Department of the GPHC administered and managed all Project finances.

Although the CCF is a CIDA funded program, the Public Works and Government Services Canada (PWGSC) was the government body responsible for administering the finances. The PWGSC was responsible for the following:

a) Preparation of the Contribution Agreement;

b) Monitoring the Project progress;

c) Receiving and processing request for advances/payments;

d) Providing and controlling the CCF financial inputs to the Project and ensuring that these funds are used in accordance with the Contribution Agreement;

e) Receiving and approving progress and financial reports.

The Project Manager of the Caribbean Canadian Cooperation Fund was designated as the PWGSC Representative.

This management structure created several difficulties for Project implementation. The CCF project manager viewed the subcontractors, not as the project champions that they were, but as hired consultants. On this basis, the CCF project manager required the project coordinator to be accompanied by the GPHC project manager or his representative for all meetings. This decision essentially eliminated any consultation between the project coordinator and CIDA. Since the project coordinator was and remains the main initiator and driving force of the Project, while being relatively inexperienced with CIDA and PWGSC procedures, this impediment was a source of continual inefficiency and resulted in corresponding delays in the project operations.

A number of errors in calculation occurred in the development of the Contribution Agreement (CA) and its Amendments. Errors in calculating in-kind contributions are dealt with in **4.1.6.** While these had no direct impact on the Project activities, they are an example of the problems associated with PWGSC control of CCF financial inputs to the Project. There were, however, many errors that did have consequences for the Project. In the fall of 2009, when critical disbursements were required for the 2^nd^ cohort of KOL course fees to IIWCC, there were significant delays in advances. This required approaching MOH to fund these activities, although they had been budgeted in Amend #2. Calls were made to PWGSC, but no advance was forthcoming. The bureaucratic impediments referred to above made communications even more difficult. When the advance was finally provided, it was inadvertently deposited into the wrong account! This further delayed access to the funds. It may be important to review the training provided to GPHC Finance Department employees responsible for the funds from CCF or PWGSC.

Communication for non-financial issues between CIDA or designates and the Project could also have been improved. CIDA Canada did not indicate that there was a CIDA support unit in Guyana to assist CIDA funded projects. Only after arriving in Guyana in March 2010 (in preparations for the Phase 2 project) and contacting CIDA, was Dr. Ostrow informed about the Programme Support Unit. This extremely useful resource ought to have been accessed during Phase 1 and the development of the Phase 2 proposal. An example of this lack of guidance is shown by the fact that exit taxes were regularly paid by Canadian visitors on departure from Guyana, an unnecessary expense according to agreement between the governments of Canada and Guyana. Dr. Ostrow spoke with CIDA Guyana on only one occasion during the entire Phase 1 Project, in January 2009, concerning possible funding sources for Phase 2. On the basis of this conversation, the successful Phase 2 proposal was submitted to the Partnership Branch of CIDA.

**3.2. Activities**

**3.2.1. Activity 1: Prerequisite Conditions - Functioning DFC/Project Office**

Although not all of the activities in this section were funded by the Project, they were all considered necessary prerequisites to the successful completion of the Project’s technical assistance activities.

**Activity 1.1: Infrastructure**

The Project rented necessary office furniture and office equipment. Other furniture and repairs were supplied by GPHC carpentry and plumbing departments.

**Activity 1.2: Training of secretary**

The first project secretary was hired in March 2008, but quit after less than 2 months employment. A second secretary was hired in late July 2008 and worked continuously until end of March 2010. A replacement was trained to substitute for various sick and leave periods. A third secretary has recently been hired. The main secretary was trained in required duties by the project coordinator using VOIP Elluminate® software donated by the Pan American Health Organization. She also received advanced computer training paid by the Project. The project coordinator certified her competence and will monitor the activity of the third secretary.

**Activity 1.3: Financial accounting & templates**

It became clear that the finances would be managed by the Finance Department of GPHC. Therefore this activity was not required.

**Activity 1.4: Outfitted DFC**

The DFC became a functional unit in July 2008 with the first Canadian visit and has continued to function well throughout all months of the Project. Minor additions to improve the layout and functionality were made during various visits.

**Activity 1.5: HbA1C testing**

With the assistance of the project coordinator and the advice of Canadian laboratory professionals, GPHC acquired accredited HbA1c equipment in March 2010. The delay in acquisition of this capacity resulted from earlier acquisition of inappropriate laboratory equipment. Testing began in April 2010. No data is yet available but external validation of the procedures will be required.

**Activity 1.6: Footwear source**

As indicated in **2.3.2.c)**, sustainable and cost-effective Plantar Pressure Redistribution (PPR) devices were chosen and, with logistical support from the project coordinator, the DFC was adequately supplied with products throughout the Project. Whether long term procurement can be transferred to GPHC responsibility is not yet clear but this will be a requirement of the Center of Excellence designation.

**3.2.2. Activity 2: Screening in Medical Diabetic Outpatient Clinic**

Screening for high risk status at GPHC takes place in the medical diabetes outpatient clinic. This weekly clinic, with a patient population of 2000, is over-loaded with patients (200-300 per clinic) and under-supplied with physicians. The Project had limited buy-in by the physicians in this clinic. One doctor, who led the work of the clinic until September 2008, successfully encouraged her colleagues to screen 902 patients (36/ week). In the ensuing 18 months, only another 350 patients were screened. Many attempts were made to solve the screening problem, but met with little success. Eventually screening relied on the DFC nurse attending the diabetic medical clinic to screen patients each week.

The situation was made worse, as construction of new facilities hampered screening even further and impinged on the space. These events have been reported in the subcontractors’ quarterly reports. Recently new leadership at the medical diabetic clinic raises the possibility of an improvement. The subcontractors consider that screening has been the least successful of the Project’s initiatives. The Project provided a patient education DVD on foot care to be shown in the clinic waiting room as an educational tool. For maintenance of the Center of Excellence status of the DFC, other personnel will need to have training in the screening tool for the high risk foot.

**Activity 2.1 60 Second Screening Tool**

This tool, adapted for easy usability to the low-income country context from a screening procedure by Dr. Shane Inlow, was introduced in April 2008 and has been used continuously throughout the Project. It has proved to be very useful as it identifies the 47% of patients with diabetes who are at high risk of developing a DFU. The identified high risk individuals need more intensive foot care and footwear education and monitoring. The screening tool implementation can reduce the overall workload. It also identifies patients with early ulcers who require treatment. It has been adopted by the MoH and will form the basis of screening for Phase 2.

**Activity 2.2 Screening data spreadsheet**

A data collection Excel spreadsheet was introduced in April 2008 and has been extremely useful for collecting patient data.

**3.2.3. Activity 3: DFC Leadership and Interprofessional Diabetic foot teams**

Leadership of DFC by the KOL team is considered to be one of the key factors in meeting all Project objectives and providing sustainable leadership for diabetic foot care beyond the Phase 1. To achieve this, the KOL team requires advanced training at the IIWCC (**see 3.2.5. below**) and also is required to be involved daily in diabetic foot care. The 1^st^ cohort consisted of a doctor, nurse, physiotherapist and a diabetes educator from the MoH (F/M=3/1). The first three persons work regularly at the DFC; the last comes once per week, but has been a crucial community role, particularly as Phase 2 is being developed.

The 2^nd^ cohort, which was selected in September 2009 under the terms of Amend #2, consists of a doctor, two nurses and a rehab assistant (F/M=3/1). Two of the four KOLs work regularly at DFC and one works at another clinic in region 4 which will become a regional DFC in Phase 2. They have all committed to participating in training other health professionals. Because of attrition from this important group, (the lead physician will be leaving in December 2010, although he will participate in Phase 2 training), it is important to maintain a pool of adequate members. To this end a 3^rd^ cohort will be selected under the terms of Phase 2 in July 2010.

Training of all health professionals in the Project has been carried out on the interprofessional model, because this is the evidence-based and most appropriate model for diabetic foot care. Collaborative care optimizes patient outcomes and acts as a physician extender with increased roles for both nursing and rehabilitation specialists. This interprofessional training has empowered nurses and broken down clinical hierarchies.

**3.2.4. Activity 4.1: Training visits by Canadian specialists**

Three training visits by Canadian foot specialists were planned in Amend #1 and these were all carried out in July 2008, January 2009 and July 2009. Various educational strategies were used including: interactive group sessions, skill training and hands-on clinical experience at the DFC. The core of information was provided in a 5 day session. An attempt was made during the 1^st^ and 2^nd^ visits to build on the experience of the trainees, but the number of new trainees made this difficult. In total 50 health care professionals were trained: 25 nurses; 15 doctors; 9 rehab specialists; 1 diabetes educator (F/M=1.5). The training experience was evaluated using a subjective opinion form. The response was overwhelmingly positive with some suggestions for improvement. An additional training visit by Dr. Sibbald in November 2009 has been discussed in **2.3.2.d.**

As part of the educational strategies, context-specific enablers for the key components of the Wound Bed Paradigm (the clinical assessment core of the Best Practices) were produced and disseminated to all trainees.

**3.2.5. Activity 4.2: International Interprofessional Wound Care Course (IIWCC)**

This acclaimed course from the University of Toronto has been training wound care specialists from Canada and internationally for over 10 years. It is an eight month course with two residential weekends, nine self study modules submitted online and a selective which integrates course material into the students practice environment. The IIWCC is both a continuing education course for healthcare professionals and a graduate level course that can form a component of a Masters in Community Health. Dr. Sibbald is the course director.

The subcontractors consider that the IIWCC is a very important component of the development model, which provides the KOLs with advanced training in both clinical practice and education. The 1^st^ cohort completed the training with 75% of them receiving certificates of completion. The 2^nd^ cohort has completed the two residential weekends and is in the process of completing their modules and selectives. Travel costs and course fees have been supported by a mixture of Project funds and other sources. The Project supported the travel fees for the first cohort (Amend#1). The course fees were waived at the discretion of the course director. Amend #2 anticipated supporting the course fees for the 2^nd^ cohort. However funds were not forthcoming and as a result the course fees were paid by the MOH. The 2^nd^ cohort’s first residential weekend travel costs were paid by PAHO and the 2^nd^ residential weekend paid by the Project in lieu of the planned course fees.

**3.2.6. Activity 5: Operation Diabetic Foot Centre**

Although no performance indicators were provided for this activity in Amend #1, it is clear from **2.3.2 a-d** that the DFC has functioned throughout the Project and continues to function, making a significant contribution to diabetes care in Guyana. It is integrated into the overall provision of clinical services at GPHC. The DFC was provided with a data entry Excel spreadsheet to capture patient profiles.

**3.2.7. Activity 6: Assessment Tools**

The tools for patient assessment were developed using a collaborative process between Canadian specialists, the KOLs and interprofessional teams. These tools underwent two iterations and were finally installed in January 2009. They comprise:

- Initial assessment forms for high risk non-ulcer patients,
- Initial assessment forms for patients with ulcers,
- Progress forms, used both in the DFC and on the surgical wards,
- Specific rehab form to capture PPR recommendations.

While these forms have limitations as far as data collection; they have been very useful clinically.

**3.2.8. Activity 7: Amalgamation with National Diabetes Program**

From the initial planning through the implementation of the PoA, the subcontractors have paid attention to situating Project activities inside the public health system of Guyana and collaborating with the appropriate Ministry of Health section, the Chronic Diseases Unit, at all stages. All facilities, program components and human resources are integrated into the MOH Program for the Control and Management of Type II Diabetes. This is a fundamental Project principle and it has been implemented.

The first activity with this collaboration was the participation of the Canadian specialist team in the editing of MOH educational material on the diabetic foot. As a result, the MOH has produced two pamphlets as provider and consumer resources.

The collaboration with MoH deepened throughout the course of the Project and has culminated in the Guyana Diabetic Foot Project – Phase 2, which will regionalize community-based diabetes care throughout 6 administrative regions in Guyana comprising 90% of the population. The Minister of Health for Guyana was strongly supportive of the Project and met with the Canadian visitors on every visit.

**3.2.9. Activity 8: Continuing education in Guyana**

This project moved Continuing Medical Education (CME) to Continuing Education (CE) that included Continuing Professional Education (CPE). For the first time, doctors, nurses and rehabilitation specialists participated in the educational program and attended activities together; promoting a better understanding of each professions expertise and promoting increased collaboration. Many members of the Interprofessional team attended lectures and other presentations that were targeted for the medical community.

While two workshops were planned, only the November 2009 workshop, described in **2.3.2.d,** was actually carried out. However 5 CME lectures on diabetic foot care to over 200 participants were given during the course of the four training visits, including an address by Dr. Sibbald to the 2^nd^ Guyana Scientific and Medical Conference in November 2009. As well, a CME group session was given at the West Demarara Regional Hospital (region 3) in January 2009. A further group session on nurse empowerment for GPHC nurses was presented in July 2009.

**3.2.10. Activity 9: Post-project analysis and dissemination**

No post-project statistical analysis has been carried out. Funds for this line item were diverted to allow travel costs for the 2^nd^ cohort IIWCC.

The Project results have been widely disseminated in diabetes and wound care forums.

Two papers on the Project were published in journals:

1. Ostrow B, Martin C, Rambaran M. **Clinicians work to enhance diabetic foot program in Guyana.** Advances in Skin & Wound Care 2008, (12):640-1
2. Sibbald R, Woo K, Ostrow B. **Preventing amputations: the need for screening, diagnosis and treatment of diabetic foot complications in Guyana, South America.** Journal of World Council of Enterostomal Therapists 2008, 28(2):34-6.

The following presentations were given on Project results:

1. May 2009, Bethune Roundtable on International Surgery, Toronto, Canada
   1. **Sixty second screening identifies persons at risk for diabetic foot ulcers – oral presentation, Brian Ostrow**
   2. **International Collaboration in Guyana to Reduce Amputations in Persons with Diabetes – oral presentation, Gary Sibbald**
2. **June 2009, Annual conference, Caribbean College of Surgeons, St. Kitts**
   1. **Early impact of Guyana Diabetic Foot Project on major amputations at GPHC – oral presentation, Carlos Martin – won prize**
3. **October 2009, 20^th^ World Diabetes Congress, Montreal, Canada**
   1. **Sixty second screening identifies persons at risk for diabetic foot ulcers – poster, Brian Ostrow**
   2. **International Collaboration in Guyana to Reduce Amputations in Persons with Diabetes – poster, Brian Ostrow**
   3. **Early impact of comprehensive diabetic foot centre on diabetes-related amputation rates at Georgetown Public Hospital Corporation, Guyana – poster presentation, Brian Ostrow**
4. **November 2009, Canadian Association of Wound Care annual conference, Quebec City, Canada**
   1. **Satellite symposium comparing wound care in Guyana with Ontario – Gary Sibbald**
5. **May 2010, European Wound Management Association Conference, Geneva Switzerland**
   1. **Sixty second screening identifies persons at risk for diabetic foot ulcers – oral presentation, Kevin Woo**
6. **June 2010, Canadian Public Health Association Centennial Conference, Toronto, Canada**
   1. **The Guyana International Collaboration Model to Reduce Amputations in Persons with Diabetes** – oral presentation, Brian Ostrow
7. June 2010, Annual Conference, Caribbean College of Surgeons, Georgetown, Guyana
   1. **Update of the Diabetic foot programme in Guyana** – oral presentation, Carlos Martin

**3.3. Resources utilized and responsibilities**

**3.3.1. Subcontractors**

The Project engaged two volunteer subcontractors to lead the Project:

1. Project coordinator – Dr. Brian Ostrow. Dr. Ostrow had overall responsibility for Project coordination and logistics, including the preparation of Project reports. His contributions were entirely voluntary, except for travel expenditures to Guyana. His in-kind contribution is calculated at over $150,000CAD.
2. Project team leader – Dr. Gary Sibbald. Dr. Sibbald had responsibility for mobilizing Canadian foot experts, and was instrumental in giving rise to the Plan of Action and interprofessional and key opinion leader models which guided the Project. Dr. Sibbald contributed 81.5% of his Project time in in-kind hours and the rest of the team volunteered 63% of their time. **See 4.6.1.**

The conclusion of this Report is that both sub-contractors performed their responsibilities to the best possible extent and were instrumental in making the Project a success.

**3.3.1.1. Hiring process**

There was no competitive hiring process. Given the background of how the Project was conceived and developed, no one other than Dr. Ostrow could have been considered for the project coordinator position. As far as Dr. Sibbald is concerned, given his reputation and renown as an international wound care leader and his experience in education and interprofessional team development, it is hard to imagine a more competent team leader.

**3.3.2. Stakeholders**

1. Georgetown Public Hospital Corporation was the designated Lead Organization. In general, GPHC management and staff were very committed to the Project and gave generously of their time and effort. An excellent feeling of collaboration and comradeship was developed between the Canadian and Guyanese teams. GPHC management made major contribution of resources, human and material, to the successful completion of the Project. There were, however, numerous instances of delay and what appeared to be disorganization. The subcontractors believe these are a reflection of problems with “capacity” which exist throughout the Guyanese public service. If these problems did not exist, no doubt external support from countries, like Canada, would not be necessary. The Guyanese public sector would benefit from capacity building projects.
2. Chronic Disease Unit, Ministry of Health is the Ministry department most directly concerned with diabetes care. Project leaders very quickly made contact with the Dr. G. Krishendat, Unit director. The diabetes educator associated with this Unit became a member of the 1^st^ KOL cohort. The collaboration between the Project and this Unit has given birth to the Phase 2 Project. The MOH made a major contribution to the Project through purchasing all PPR redistribution supplies ($9506.74CAD) via a procurement process referred to in **2.3.2.c)**. Reimbursement of the project coordinator was delayed however. The MoH also supported the 2^nd^ KOL cohort through the IIWCC course fees. The subcontractors thank MoH for these contributions.
3. The New Women’s College Hospital (WCH) in Toronto, Canada was designated in Part C1 of the Contribution Agreement as the source of in-kind contributions of $15,000CAD. However, Woundpedia, [www.woundpedia.com](http://www.woundpedia.com/) , incorporated as a not for profit organization based on the proceeds from the World Union of Wound Healing Societies meeting in Toronto June 4-8, 2008, is a more appropriate source to account for the in-kind contributions of Canadian volunteers since not all volunteers were employed by the WCH.
4. Caribbean Canadian Cooperation Fund was the CIDA fund under which the Project functioned. As explained in this Report, the authors had very little contact with CCF officials.
5. Public Works Government Services of Canada administered the Project funds.
6. Pan American Health Organization is very active in supporting diabetes and chronic disease capacity development in the public health system in Guyana. Contact between the Project and PAHO occurred towards the end of the Project, but resulted in support for the travel costs of the 2^nd^ KOL cohort to the first IIWCC residential weekend. The subcontractors thank PAHO for this contribution and aim to increase this collaboration during Phase 2.
7. Persons with diabetes (PWD) in Guyana are the ultimate target and beneficiaries of all Project activities. The Project aims to deliver patient-centred care and PWD appeared to be very appreciative of the services provided.

**4. Analytical Review of the Project**

Much of the analysis of specific Project activities has been included in the **3. Summary Description.** What will be included here are more general reflections of the Project’s achievements and weaknesses.

**4.1. Project Rationale and justification**

- Are diabetic foot complications a significant burden to the public health system in Guyana and a serious threat to its adult population?
- Were the current management systems in place in Guyana at the time of Project initiation inadequate to deal with this situation?
- Were the Project activities a step towards changing these conditions?

These questions speak to the rationale and justification for the Project. Notwithstanding the limited data available, the answers to these three questions, based on the data provided in this Report, are clearly affirmative in the opinion of the authors.

**4.2. Results**

Since a summary of the Results is presented in the **2.2, 2.3** and **3.2** above, only a brief review focussing on analysis will be presented here. The biggest overall weakness with regard to the Results is that indicators were not defined for the various levels of developmental outcomes at the start of the Project. This occurred because of the modifications to the project to optimize outcomes based on the conditions in Guyana and the local education and clinical practice needs that required a new infrastructure. The Results Based Management of this project will be enhanced in Phase 2 with increased project coordinator and project leader experiences in Guyana. We would also appreciate a mechanism of receiving guidance from CIDA.

**4.2.1. Expected versus Actual Results**

Referring to each level of developmental outcome:

**a) Goal** – the authors believes that there is justification in concluding that the Project “***improved the overall health of persons with diabetes in Guyana by developing an enhanced diabetic foot program at the Georgetown Public Hospital Corporation (GPHC)****”.*

**b)** **Objectives/developmental outcomes** - the authors believes there is justification in concluding that the Project achieved success in implementing all four developmental outcomes. Even when the outcome was not completely to the satisfaction of the subcontractors, eg screening, the format and components of a successful system were developed by the Project. Probably the biggest risk to the sustainable functioning of a comprehensive diabetic foot program in GPHC is the maintenance of a cadre of KOLs, as a result of outmigration and attrition of trained staff. For this reason the subcontractors believe that the Phase 2 project, 3^rd^ cohort of KOL and the ongoing support by Canadian experts over the next 3 years are necessary to solidify the achievements of Phase 1.

Furthermore the authors believe these objectives were appropriate to build local Guyanese capacity for the prevention and management of diabetic foot problems. Prevention is paramount and is achieved through screening for risk status and patient education in foot care and foot wear. Treatment should be comprehensive and based on evidence, such as the Best Practice Recommendations of the Canadian Association of Wound Care. Plantar pressure redistribution is a vital care modality which requires specific training and resource mobilization. Capacity to educate the Guyanese health community on the principles of diabetic foot care is the ***sine qua non*** of sustainability.

**c) Activities/outputs** – the authors believe there is justification in concluding that the Project achieved most if not all of its outputs. It is too early to tell whether the new HbA1c testing will work adequately, but there is every reason to believe it will. Screening remains a concern, but there is reason to believe that new leadership in the medical diabetes clinic will improve the situation. The assessment tools do not lend themselves to data analysis, to the full extent desirable, and will be altered accordingly for Phase 2. The question of procurement and cost of PPR devices are issues, but again the format and components of a system have been delivered. It will be up to GPHC leadership to implement them. It may be that a cost recovery system will be necessary for PPR devices. Aside from these deficiencies, the Project’s main technical assistance activities were actually over-performed compared to those anticipated in the PoA.

**4.2.2. Expertise Utilized**

The expertise, of the Canadian wound care expert team, led by Dr. Gary Sibbald, needs to be recognized first. This group of doctors, nurses, chiropodists successfully modeled interprofessional care, adapted Canadian practice to the Guyanese conditions and delivered wound care education in a collaborative fashion.

The Best Practice Recommendations for the Prevention, Diagnosis and Treatment of Diabetic Foot Ulcers, published by the Canadian Association of Wound Care, were adopted as the theoretical and scientific basis of clinical activity and training. These Recommendations incorporate evidence-based guidelines for clinical care from the Registered Nurses Association of Ontario (2004, 2005) on the Diabetic Foot and Anti-infective Guidelines on Community-acquired Infections (2005) into a practical bedside enabler.

**4.3. Intellectual property**

The enablers, assessment tools and data spreadsheets created for this Project have the following dispositions:

1. 60 second screening tool – this has been copyrighted by Dr. Gary Sibbald but can be used free of charge by the DFC and MOH.
2. All other assessment tools, enablers and data spreadsheets can be used free of charge by the GPHC and Ministry of Health, Guyana. They will be adapted to be used in Phase 2 and may be used or adapted for training in similar settings.

**4.4. Political considerations**

There were no specific political considerations relevant to the Project.

**4.5. Gender Equality issues and results**

The Project paid serious attention to gender equality and kept gender disaggregated data on all Project participants. These have been documented in this Report. While no gender specific training was carried during this Project, its value is recognized and will be implemented during Phase 2.

It is clear that men do not present for screening. There must be a gender specific focus to encourage men to come for screening.

The inter-professional model, by its nature, promotes gender equality. It empowers nurses who are by and large female. It also breaks down clinical hierarchies which have a gender component. A reflection of this is the position of the head nurse at the DFC. She is a woman, who has blossomed under the Project. Her background is as a registered nursing assistant (registered practical nurse in Canadian terms), yet she was able to pass the IIWCC, a graduate university course! Now she is considering studying for her registered nursing degree.

**4.6. Disbursements**

The Project Budgets and their Amendments together with the actual cumulative expenditures for each line item to date are presented in **Table 6**.

**Table 6**

| **Line item** | **Total Approved Initial Budget** | **Amend # 1** | **Amend # 2** | **Actual cumulative expenditures** | **Variance** |
| --- | --- | --- | --- | --- | --- |
| **Project Management** | 3000 | 3750 | 5000 | 5,396.07 | +396.07 |
| **Technical assistance** | 20000 | 17100 | 7900 | 12,238.27 | +4338.27 |
| **Travel cost for applicant personnel** | 500 | 18720 | 25400 | 27405.91 | +2,005.91 |
| **Travel cost for sub-contractors** | 29836 | 36430 | 37200 | 36,806.17 | -393.83 |
| **Rental of Equipment** |  | 8000 | 7600 | 7,327.34 | -272.66 |
| **Administration (office costs)** | 9600 | 1600 | 1500 | 1,449.59 | -50.41 |
| **Dissemination** | 3000 | 3000 | 4000 | 5,692.21 | +1,692.21 |
| **Overhead (10%)** | 6,593 | 8,860 | 8,860 |  |  |
| **TOTAL** | **72,529** | **97,460** | **97,460** | **96,315.56** | **-1,144.44** |

With Amendment #1 the Project Budget increased substantially to support the new activities planned by the PoA. Amendment #2 delayed the Project completion date to May 31, 2010 and shifted unused funds from technical assistance to support the 2^nd^ cohort of KOLs.

The variances are explained as follows:

1. **Project management:** The increased salary requirements of the Project secretary resulted from the increased length of the Project.
2. **Technical assistance:** As explained above, Amendment #2 predicted that there would be savings in technical assistance that could be applied to the travel costs/course fee costs for the 2^nd^ cohort of KOLs. The technical assistance line item was reduced accordingly. While there were significant savings compared to the technical assistance envisioned in Amendment #1, the total cost of technical assistance was underestimated by Amendment #2 resulting in a significant variance.
3. **Travel cost for applicant personnel:** The cost of sending the 2^nd^ cohort KOL to the 2^nd^ residential weekend of the 2009-10 IIWCC is reflected in this line item, resulting in a small variance from budget.
4. **Travel cost for sub-contractors:** This line item includes the additional training visit by Dr. Sibbald in November 2009, an unbudgeted but very important activity as explained in **2.3.2.d.** Despite this addition the variance from budget is small.
5. **Rental of equipment:** This line item was budgeted relatively accurately.
6. **Administration (office costs):** This line item was budgeted relatively accurately.
7. **Dissemination:** This line item was significantly over-budgeted despite the extensive dissemination activities undertaken.

**4.6.1. In-kind contributions**

The Contribution Agreement required in-kind contributions from:

- GPHC - $20,000.
- Woundpedia- $15,000.

The GPHC contributions are based on in-kind hours from the project coordinator. At $100/hr, working 3 hours/day in Canada over 2 years (March 19, 2008 to March 19, 2010), this represents a contribution of $150,000, well beyond the $20,000 required.

The Woundpedia contributions are based on the non-reimbursed hours of the Canadian foot specialists. This is represented in the **Table 7** below. It is clear that WoundPedia has contributed well beyond its required in-kind contributions as well.

**Table 7 In-kind hours for Woundpedia** volunteers

| Category | Working days in  Guyana | Honorarium  /working day | Hourly rate | Hourly  in-kind contribution | Daily  in-kind contribution | Total  in-kind  contribution |
| --- | --- | --- | --- | --- | --- | --- |
| MD | 27 | $150 | $100 | $81.25 | $650 | $17,550 |
| Nurses/  chiropodist | 54 | $150 | $50 | $31.25 | $250 | $13,500 |
| **Total** |  |  |  |  |  | **$31,050** |

Examining the CA and associated Amendments #1 & #2, it is clear that an error occurred in documenting the in-kind contributions. While the designated in-kind contributions totalled $35,000 ($20,000GPHC and $15,000 Woundpedia), when responsibility is assigned for this in Part C Article 2, only $20,000 is noted. This was changed in Amend #1 to $34,500. Presumably this was because the increased budget (CIDA contribution = $97,460) required an increased in-kind contribution. This whole amount was then inserted into the Woundpedia in-kind contributions in the Amended Budget and the $20,000 of in-kind contributions of GPHC were not recognized. However the initial $35,000 in-kind contributions were more than adequate to account for the required 3:1 in-kind contributions (97460/35000=2.78). This error then was continued into Amend #2. Although these errors had no practical consequence on the Project finances, they are a good example of the problems associated with PWGSC control of CCF financial inputs to the Project.

**4.7. Scheduling and logistical difficulties**

There were no real scheduling difficulties during the Project. The project coordinator kept all stakeholders apprised of the scheduled activities and they all participated to the fullest extent possible. Because of various time constraints the Canadian visits were shorter than they had initially been planned. This did not seem to have a deleterious effect on training. The re-iteration of approach through the 3 visits was extremely valuable.

As noted in **3.3.2 (1)** there were numerous logistical difficulties, which might be expected in a project such as this. These were overcome to the extent possible by perseverance.

**4.8. Knowledge sharing results**

As can be seen from the list in **3.2.10**, the experience of the Project has been richly shared with the broader medical and public health community. The most concrete result of knowledge sharing, to date, comes as a result of Dr. Sibbald’s work to hold IIWCC in Africa, through collaboration between Stellenbosch University and the Dalla Lana School of Public Health at the University of Toronto. As a result, African students are introducing the 60 second screening tool in their practice contexts. There are also potential collaborations with the International Diabetes Federation’s Step by Step project in the Caribbean.

**4.9. Synergy**

There are many CIDA funded Canadian NGOs at work in Guyana. We have recently made contact with Health by Empowerment [www.healthbyempowerment.ca](http://www.healthbyempowerment.ca/) which has been training community health workers in diabetes care in Guyana. This experience will be integrated into the Phase 2 project.

PAHO is actively collaborating with the MoH in Guyana. They have specific projects around diabetes and will be conducting what may be the first comprehensive prevalence study of diabetes in Guyana. Phase 2 will collaborate with PAHO to the fullest extent.

**5. Conclusions**

Thanks to the CIDA grant and the health care professionals in Guyana and Canada, the Guyana Diabetic Foot Project – Phase 1 at the Georgetown Public Hospital Corporation has:

- provided a dedicated program for the prevention, diagnosis and treatment of persons with diabetic foot ulcers
- trained 8 key opinion leaders in evidence-informed interprofessional practice for optimal diabetic foot care (Canadian interprofessional team visits, local educational program including interactive lectures, workshops, clinic preceptorships and the International Interprofessional Wound Care Course at the University of Toronto for Key Opinion Leaders, individual mentoring)
- developed the first Interprofessional practice model in Guyana, South America linking physicians, nurses and rehabilitation specialists for improved patient care education and research and training 65 health care professionals
- implemented an educational model to link education to improved patient outcomes
- empowered several female doctors, nurses and rehabilitation specialists for leadership roles
- developed a 60 second screening program for prevention of diabetic foot complications
- enhanced care of the diabetic foot by introducing plantar pressure redistribution devices into everyday diabetic foot care in Guyana through the training of rehabilitation specialists in Guyana
- appraised current diagnosis and treatment of the infected diabetic foot and improved antibiotic regimes and treatment outcomes including more conservative debridement techniques
- reduced the amputation rate by 46% for persons admitted to the GPHC with acute diabetic foot ulcer complications in the context of ongoing increases in diabetic foot admissions to the hospital.

The Disbursements have been presented and the variances from budget have been discussed. The GPHC and WCH have both fulfilled their in-kind contribution responsibilities.

The subcontractors wish to thank all the Canadian and Guyanese participants in the Project. The results presented in this Report could not have been achieved without the dedication and hard work of everyone involved. They show what can be accomplished in a short time and without a lot of money, when effective collaboration is based on sound principles. We are very proud to have participated in this Project. We believe CIDA and the Government of Canada have received very good value for their investment.

**Submitted: June 14, 2010**

**Brian Ostrow MD, FRCSC – project co-ordinator**

**R Gary Sibbald MD, FRCPC (Derm & Int. Med.), M.Ed. – project team leader**

**Sign-Off**

June 23, 2010

Signature Date

Dr. Madan Rambaran

Name (printed)

Director, Medical & Professional Services

Title
