# Supplementary material for: The Guyana Diabetes and Foot Care Project: A Complex Quality Improvement Intervention to Decrease Diabetes-Related Major Lower Extremity Amputations and Improve Diabetes Care in a Lower-Middle-Income Country
Source: PLoS Med. 2015 Apr 21;12(4):e1001814. doi: 10.1371/journal.pmed.1001814 (PMC4405371; doi:10.1371/journal.pmed.1001814)
Supplement: S2 Text — (DOC) [file pmed.1001814.s003.doc]

**CIDA Canadian Partnership Branch**

**Project No. S064802 Purchase No. 7054813**

**The Guyana Diabetes and Foot care Project**

**(Formerly the Guyana Diabetic Foot Project – Phase 2)**

**Final Narrative Report**

**March 1, 2010 – February 28, 2013**

**Table of Contents**

1. **Executive summary**
2. **Summary of Project goal and objectives, activities, and expected results**
3. **Main risks and critical assumptions identified**
4. **Analysis of gender equality issues and results**
5. **List of goods purchased**
6. **Intellectual property**
7. **Description of the collaboration between the project’s various partners**
8. **Sustainability of results**
9. **Analysis of the main lessons learned in the process of Project implementation**
10. **CAGS cost-sharing obligations**
11. **Explanation of Variances**
12. **Executive summary**

A frank assessment of the 3 years activities of the Guyana Diabetes and Footcare Project – Phase 2 shows a mixture of positive results and some significant disappointments. On the positive side:

- The Project model, using international collaboration to implement longitudinal, inter-professional and multi-faceted (including on-job training) educational strategies based on context-specific and evidence informed best practices combined with capacity building including infra-structural improvement and healthcare system change, is an effective approach to translate knowledge into practice in a resource-constrained country such as Guyana.
- The maintenance and expansion of the existing interprofessional team of trainers (key opinion leaders (KOL)) through attendance at the International Interprofessional Wound Care Course in Canada (Project funded 3 new cohorts – 7 persons) and an online diabetic educator course through the Canadian Michener Institute facilitated in Guyana (10 persons; 7 new) – a potential team of 19 trainers.
- Through training, infrastructure improvement and health system change, the Project built innovations in comprehensive (both prevention and management) diabetes, diabetic foot ulcers and blood pressure care onto the existing structure of the publically funded health care system in designated regions. These innovations included:
  - Iterative interprofessional training of 275 health care professionals (doctors, medex, nurses, rehab assistants, community health workers) in 97 facilities by local KOL team
  - Application of the 60 second foot screening tool (3452 screens) to identify patients at high risk of developing foot ulcers (65% at high risk).
  - Development of 7 regional diabetic centres by renovating existing facilities and supplying toolkits and essential equipment to provide specialized diabetic foot care for high risk patients and those with ulcers.
  - Development of assessment tools and enablers (guides for clinical practice) for persons with diabetes, high blood pressure and foot ulcers.
  - Identification and approval by Ministry of Health (MoH) of indicators for data collection, development of data collection processes, tools and human resources
  - Development of monitoring and evaluation processes including regional KOL mentors; surveillance supervision tools, practice guidelines and integration with the MoH Inspectorate
- Support for HbA1c testing with an accredited machine at the national referral hospital and the development of regional testing using an unaccredited process.
- The positive clinical results of Phase 1, including a 52% reduction in diabetes-related major amputations at the national referral hospital, have been sustained throughout Phase 2.
- A greater understanding of the unique gender ratios of Type 2 diabetes in Guyana (F/M = 2.09) has been developed which confirm preliminary international findings.
- Baseline values of prevalence of diabetic foot ulcers (10% of persons screened) and HbA1c levels in the population have been determined.
- New information about the association of diabetic foot ulcer and its attendant complications such as amputations with the male gender.
- The Ministry has solicited and received input from the Project on its Chronic Non-Communicable Disease (CNCD) Program (**see 8b**).
- The Project received the 2013 *Journal of Wound Care* Award for Best Research from a Developing Country. Also won two poster awards: at WUWHS meeting in Japan September 2012 and Clinical Symposium of Skin and Wound Care, Las Vegas, 2012

On the negative side:

- The collaboration between the Project and the MoH was not consistently effective during the life of the Project. While there are a number of reasons for this, outlined in **7c**, the most important was the absence of a Ministry coordinator for chronic diseases with whom the Project could engage. This absence, from August 2010 to January 2013, also limited the attention paid to chronic diseases generally in the MoH. The consequences of ineffective collaboration were:
  - Significant delay in completion of rDFC renovations – three facilities were not completed until the last months of the Project
  - Significant delays in resolving the mechanism of data sharing.
  - Significant delays in many other Project innovations e.g. implementation of assessment tools in regional facilities
  - Abandonment of certain activities, vis. patient questionnaires

The overall consequence of ineffective collaboration is that, while the framework of integrated comprehensive care has been developed, contact with the anticipated numbers of persons with diabetes has not occurred. Therefore, rather than a total of 14,565 persons with diabetes (PWD) benefitting from the Project, as predicted; only about 7567 persons were contacted and included in the database and the quality of this contact is variable. This state of affairs was recognized by March 2011 and reported in the 2^nd^ interim report. Project partners concluded early on that in order to resolve the situation and bring the innovations into full effect, further collaboration between external partners and the MoH would be necessary. The hiring of a coordinator for Chronic Non-communicable Diseases in January 2013 and the development of a Ministry draft *Strategic Plan 2013-2020: Integrated Prevention and Control of Non Communicable Disease in Guyana*, which recognizes Project innovations, are two recent very positive developments which, in the view of this author, justify further collaboration. As well, the expressed interest taken by the Banting and Best Diabetes Centre of the University of Toronto in leading a future collaboration provides the basis for a strong external partner.

1. **Summary of Project goal and objectives, activities, and expected results**

The Project goal and objectives were as follows:

**Project Goal:** To create a sustainable, comprehensive, national diabetic foot care program in Guyana and thereby improve the health of 37,600 (2007 estimate) (Men and Women) persons with diabetes (PWD) in Guyana.

| % Persons tested with HbA1c>9%  **Baseline only** | 38% of 4588 patients with first tests > 9%  HbA1c: Average 8.56%  Females - 8.66% Males – 8.31%  Patients with foot complications – 9.37% |
| --- | --- |
| Prevalence of foot ulcers in PWD  **Baseline only** | 372 patients of 3422 had an active ulcer = 10%  Females: active ulcer 255 out of 2377: 10.7%  Males: active ulcer 108 out of 944: 11.4% |

For diabetes-related major amputations we can report:

| Diabetes-related major amputation numbers and rate | **52% reduction from pre-Phase 1 level sustained through December 2012**. ~ 50 limbs saved each year  68% reduction of GPHC inpatients with Diabetic foot complications receiving major amputations |
| --- | --- |

The **Project Objectives** were expressed as Intermediate and Immediate Outcomes and Outputs and are reported in the Final Report on Performance. We can make the following comments on specific Outcomes and Outputs:

| **Outcome or output** | **Comments** |
| --- | --- |
| **INTO100**-353 HEALTH WORKERS IN REGION 2,3, 4 5,6,10 BY 2012 ENGAGING IN TRAINING ON COMPREHENSIVE DIABETIC FOOT CARE PRACTICES | 275 health care workers were trained from 97 facilities in 2 cohorts. A decision was made to reduce cohort 2 to concentrate on on-job training. Additional medex and rehab assistants were trained inside their respective training programs. |
| **INTO201**-6000 PWDS PARTICIPATING IN COMPREHENSIVE PREVENTION AND TREATMENT PROGRAMS IN REGION 4 BY 2012 | Screening in Region 4 was limited by difficulty in engaging screening at the GPHC medical clinic. 1521 patients screened at GPHC since July 2008. 575 screened outside GPHC in Region 4.  2729 high risk or ulcer patients seen in DFC from July 2008 to Dec 2012. DFC-GPHC sees about 20 new patients with ulcers/month |
| **INTO202** - 540 CHRONIC WOUND CARE PATIENTS ENGAGING IN ADDITIONAL APPROPRIATE CWC PRACTICES AT DFC BY 2012 | Because of the burden of disease at GPHC the number of non-diabetic patients with chronic ulcers was restricted. |
| **INTO 301** - 7500 PWD IN REGIONS 2,3,5,6,10 BY 2012 PARTICIPATING IN PREVENTATIVE PROGRAMS | Total 3452 PWDs screened since Jul 2010 in all regions. Screening is going slower than expected after initial positive result. This is a reflection of the challenges the project has faced. |
| **INTO302**- 525 PWD IN REGIONS 2,3,5,6,10 BY 2012 PARTICIPATING IN TREATMENT PROGRAMS | To date 1186 patients (F/M = 1.60) with foot ulcers have been treated at project regional diabetic foot centres. Corrects data from April 2012. Over 20776 visits for dressing care. Note increase # of males with ulcers compared to screened patients |
| **INTO401** - Enhanced gender equality in project human resources by 2012 | The reality is there are more women than men in health care in Guyana and possibly in Canada as well. Attempts to correct gender imbalance being made especially in KOLs (2 males in KOL5) |
| **INTO 402** - Enhanced parity in provision of care to PWD in project regions by 2012 | F/M ratio of current project database (7451 with gender info) = 2.09. This may be a true reflection of gender ratio of diabetes in Guyana. In 2012 for the first time Diabetes Atlas 5th stated F/M=2.25 Diabetes in Guyana – this is a significant variance from world figures 0.95. Our results confirm this! |
| **INTO404**- GPHC and MOH fulfilling project responsibilities by 2012 | Proxy measure: HbA1c tests. 5115 tests to Feb 2013 up from 3289 in March 2012. D-10 unit at GPHC non-functional from April 2012 – Dec 2012. Issue of speed of repair. Progressive increase in HbA1c testing |
| **IO100**- INCREASED DFC CAPACITY TO TRAIN HEALTH WORKERS IN REGION 2,3,4,5,6,10 BY 2013 IN COMPREHENSIVE DIABETIC FOOT CARE PRACTICES | Completed satisfactorily  Cohort 2 trainings and trainees reduced to concentrate on Stream 3 on job training. Training is iterative |
| **IO200**-CONSOLIDATE CLINICAL CAPACITY FOR DIABETIC FOOT PREVENTION AND TREATMENT IN REGION 4 BY 11.2010 | Outcome completed  a) Final decision re rDFCs in R4 – 2 facilities  Renovation completed at Grove HC in January 2013 |
| **IO201**-CONSOLIDATE CLINICAL CAPACITY OF DFC AS COE - ADDITIONAL APPROPRIATE CHRONIC WOUND CARE BY 2013 | DFC has stopped seeing non-diabetic patients with chronic wound problems, because of too many patients with diabetes. Outcome incomplete. |
| **IO301**- SUSTAINABLE CAPACITY TO DELIVER COMPREHENSIVE PREVENTATIVE DIABETIC FOOT PROGRAMS TO PWD IN REGIONS 2,3,5,6,10 BY 2013 | There are 67 HC/HP facilities with trained personnel in Cohort 1 & 2. Outcome completed. |
| **IO302**-SUSTAINABLE CAPACITY TO DELIVER COMPREHENSIVE TREATMENT DIABETIC FOOT PROGRAMS TO PWD IN REGIONS 2,3,5,6,10 BY 2013 | a) all 7 rDFCs operational  b) There are 67 HC/HP facilities with trained personnel in Cohort 1 & 2. Outcome completed.  R6 and R10 DFCs were not completed until February 2013 |
| **IO401** – Increased understanding of gender concerns amongst project trainees by 2012 | All Project participants trained in gender features of diabetes in Guyana |
| **IO402**- Better understanding of gender aspects of diabetes in Guyana by 12.2010 | PAHO/MOH STEPS project not yet rolled out.  F/M = 2 for diabetes appears to be real.  Women have slightly, but significantly higher mean HbA1c levels than men (0.087 compared to 0.083). Foot complications such as amputations are associated with male gender. |
| **IO403** -Improved implementation of preventative practices by PWD in all project regions by 2012 | Patient questionnaires not completed |
| **IO 404** - More men participating in project screening by 2012 | The F/M 2.09 may be a true reflection of gender ratio of diabetes in Guyana |
| **O101 – O411** all completed satisfactorily except as indicated below: | |
| **O301** - 93 COMMUNITY HEALTH WORKERS TRAINED TO DO SCREENING AND REFERRAL AS PART OF DIABETES EDUCATION BY 2013 | Inadequate numbers of CHW chosen by MoH to be trained |
| **O403** - gender data from MOH diabetes register analysed by 12.2010 | Could not acquire data from diabetes register |
| **O410**- CAPACITY-BUILDING STRATEGIES FOR GPHC AND MOH TO FULFILL RECOGNIZED NEEDS | Project relied on MoH-World Bank Management Training Program for middle management training. The funding for this ended around June 2012 and MoH chose not to participate in any further training. Some Project trainees trained (Region 5 & 6) |

1. **Main risks and critical assumptions**

The main risks identified prior to Project implementation are identified in the table below:

| Perceived Risks/Challenge | Possible Impact on  Project Implementation & Success | Possible Strategies to Prevent or Mitigate Risks |
| --- | --- | --- |
| 1. Trained health professionals migrate out of country or move to other MOH positions | Capacity building of personnel in diabetic care will not reach anticipated levels. | - Work with local partners to factor in anticipated loss of some trained personnel by training sufficient numbers at different skill levels, particular emphasis on KOLs who can sustain a pool of trained doctors, nurses, medex, and community health workers. - Work with senior Guyanese decision makers at GPHC and the MOH to improve compensation of health workers as well as enhance professional status of trainees, leading to greater job satisfaction. Continue to emphasize the importance of maintaining financial and HR commitments. - Agreement in place with GPHC and/or MOH that the employment of project trainees will be secure. - Project encourages retention because of improved job satisfaction resulting from training |
| 1. Inability of local partners to meet project timelines | Achievement of outcomes within project timeline may not be achieved. | - Through workshops and consultations with project partners identify barriers to meeting project timelines. - Work collaboratively to develop a capacity building plan based on the local partners’ priorities. |
| 1. GPHC/MOH cannot fund all the direct patient care resources – especially HbA1c and plantar pressure redistribution (PPR) devices | Achievement of outcomes within project timeline may not be achieved | - Pay attention to project sustainability at all levels of project planning and decision-making - Capacity building for financial management - MOH to consider cost-recovery |
| 1. Unanticipated costs to rehabilitate facilities to create regional diabetic foot care centres. | Achievement of outcomes within project timeline may not be achieved | - Baseline assessment of infrastructural needs to be completed at July 2009 visit - Project can fund capital costs for infrastructure development |
| 1. Lack of MOH capacity to commit to sustain project benefits due to budgetary cuts or other reasons. | Achievement of outcomes within project timeline may not be achieved | - To date commitment has remained strong. The Project team will work closely with the MOH partners to encourage ongoing commitment. - Pay attention to project sustainability at all levels of project planning and decision-making |
| 1. Plantar- pressure redistribution (PPR) not sustained | Sustained improvements in the long-term not realized. | - Train adequate rehab specialists - Ensure sustainable inventory, restocking and purchasing capacity - MOH to consider cost-recovery - Attention paid to cost effectiveness in choosing PPR devices |
| 1. Individual beneficiaries unable to sustain improvements in their living conditions – footwear and medication costs, difficulty sustaining life-style changes | Sustained improvements in the long-term not realized. | - Ongoing awareness programs and continued contact with patients will reinforce importance of alterations to lifestyle and behaviour changes. - MOH gender-sensitive and culture-specific educational materials distributed and serve as enablers for these changes - Continue to emphasize to MOH the importance of universal free drug plan. |
| 1. Excess project salaries cause internal brain drain away from other clinical areas | Disruption of MOH human resources strategy | - All salaries/compensations will be vetted and approved by developing country partners |

Of the 8 risks identified above, numbers 3, 4, 6 & 8 were not actually problems. At present we cannot assess the possible impact of #7. Real risks included:

- Trained health professionals migrate out of country or move to other MOH positions (#1) was a very real problem. Migration out of the country of KOLs was mitigated by ongoing training of leaders including an initially unplanned 5^th^ cohort as well the training of 10 health care professionals in a Michener diabetes educator course. While the Project attempted to choose medex rather than doctors, since the latter appeared to be the main émigrés, this was not always successful. Trained personnel moving to other MoH positions, was a real problem, the extent to which was underestimated. We tried to mitigate this by stipulating that designated facilities need to be staffed by trained personnel. The long term success of this strategy is not clear.
- Inability of local partners to meet project timelines (#2) was also a very real problem. The reasons for this inability are multi-faceted and discussed in **7c**. Probably the biggest reason was the lack of an effective Ministry coordinator of chronic disease from August 2010 to January 2013. However a failure to understand Ministry procedures and an absence of same in certain situations were other reasons. This was particularly true for facility renovation where three of seven facilities were completed only in the last months of the project, despite ongoing pressure and a discussion with the Permanent Secretary and apparent agreement of approach in March 2010. Renovation problems plagued the Project as many contractors appeared unwilling to follow CIDA procedures. There is no question that this Risk constituted the main reasons for failure to meet project objectives.
- Lack of MOH capacity to commit to sustain project benefits due to budgetary cuts or other reasons (#5) was also a reason for failure to meet timelines. While the MoH always expressed commitment, there were often failures to meet timelines. The issue of data sharing is a case in point. From December 2010 – June 2011 no agreement could be achieved on data sharing until intervention by the CIDA Guyana Director and the subsequent signing of a Memorandum on Data Sharing. Data sharing was delayed and data was received on only two subsequent occasions after direct requests.
- One major risk not recognized prior to Project implementation was security. Thefts of equipment occurred at two rDFCs. While all the stolen equipment was recovered at one site; the theft in Region 10 necessitated purchase of a new computer system. Both thefts significantly delayed data entry at these sites. Once security was identified as a potential problem, all rDFC sites were secured at all possible entry points.
- The salaries for project staff initially made it very difficult to find an appropriate project manager and the salary was therefore raised to come into line with market conditions.

1. **Analysis of gender equality issues and results**

There has never been a formal prevalence study undertaken of Type 2 diabetes in Guyana. In Diabetes Atlas 4^th^ edition (DA4) the gender ratio in Guyana was estimated as F/M= 1.16. In the DA5 for 2012 in Guyana, the F/M = 2.25. This is a major change and at significant variance with the global F/M = 0.95. From discussions with DA5 epidemiologists we were informed that the estimate is based on similar conditions that are felt to exist in Belize.

All data generated by the Project was gender disaggregated. The Project data has always shown a marked female predominance in HbA1c testing and 60 second screening. The total is F/M = 2.1 for all registered patients in the database. This provides good corroboration with the new DA5 data. The tentative conclusion is that in Guyana female gender is associated with diabetes (**Χ^2^_1_= 10365, p<0.00001).** The reasons why Guyana diverges so much from global gender ratios are unexplained.

Furthermore the data 2005 – 2012 from GPHC shows an association between major amputations and male gender. In 2012 the F/M = 0.9. The analysis shows that there is an association between male gender and major amputations **(Χ^2^_1_ = 8.487, p<0.01).**

1. **List of goods purchased for the Project**

Seventy-nine thousand seven hundred and fifty six dollars ($76,783) of project funds were disbursed in line item E for equipment and renovation costs. Of this $39919 was disbursed for renovation costs on the 7 regional diabetic foot centre (rDFC) for a cost of $5702/centre. The summary of these costs is in Appendix A. Therefore, $ 36,864 was spent in Canada and Guyana on equipment. These items were either immediately transferred to MoH or have handed over at the end of the project. The invoices for these items are attached in Appendix B. They include:

1. Equipment – toolkits (including Dopplers and infra-red thermometers) and other equipment (including 3 autoclave units) to stock 7 rDFCs.
2. 3 laptops – for project manager, clerk and GPHC laboratory
3. Various office equipment – multi-function printer, laminator
4. 9 computers, surge protectors and battery back-ups for 7 rDFCs and the Health Statistics Unit at MoH (one system replaces stolen system in region 10)
5. **Intellectual property**

The project produced a number of training modules, assessment tools, practice guidelines, etc. that were handed over to the MoH, either directly or to the Institute of Health Sciences Education and are documented in Appendix C.

1. **Collaboration**

Collaboration is the key to success or failure of a project like the Guyana Diabetes and Footcare Project. The main partners and the character of their collaboration were:

1. **Canadian Association of General Association (CAGS)** As the Lead Organization CAGS had overall responsibility over project implementation. However, in practice, its main responsibility was to ensure financial accountability. Despite some challenges due to inexperience, CAGS maintained financial accountability. This is attested by the submission of all financial statements on time and their acceptance by CIDA. The day to day management of the Project was the responsibility of the project coordinator in Canada and project manager in Guyana. These two persons certainly made every effort to ensure successful implementation of project objectives and goals. However, sometimes they failed to adequately understand the procedures and requirements of the MoH. This limited effective collaboration. The 2013 Best Research from a Developing Country Award given to the project by the Journal of Wound Care attests to international recognition of the results obtained.
2. **Canadian International Development Agency (CIDA)** As donor, CIDA’s responsibility was to provide overall project guidance and support as well as financial supervision. In both these spheres CIDA fulfilled its responsibilities. In Guyana particularly, the CIDA team, including the Program Support Unit, were invaluable in providing assistance to the Project, both for understanding CIDA’s requirements as well as ensuring that successful collaboration took place between the Project and MoH.
3. **Ministry of Health (MoH)** Since all Project activities took place within the publically-funded Guyanese health care system, effective collaboration with the MoH is an absolute necessity for Project success. The successes of the Project are indicators of effective collaboration and similarly its failures are indicative of ineffective collaboration. The challenges to effective collaboration can be defined in two major classifications:
   - Failure of Project partners to understand and/or follow MoH interests, requirements and procedures: Specific instances of this type of challenge occurred around the tendering process and renovation of facilities, the recruitment of MoH personnel for overseas activity and data sharing. In some cases there did not appear to be identifiable or operational MoH procedures.
   - Lack of MoH capacity to engage with Project: The absence of a coordinator for CNCD throughout most of the Project duration has been highlighted several times in this report. The failure of MoH officials to acknowledge receipt of Project correspondence was a persistent problem.
4. **Sustainability of results**

From its inception the Project has recognized the necessity of sustaining results beyond its lifespan. The entire Project structure has been directed towards sustainability by embedding all activities inside the public health system. As such all project activities, (e.g. training, clinical best practices, facility renovations) are being implemented in the public health system. Moreover, a number of specific decisions by the Ministry indicate the extent to which transition of the Project to a Ministry programme is already taking place:

1. The Ministry’s draft *Strategic Plan* *2013-2020: Integrated Prevention and Control of Non Communicable Disease in Guyana* are based on the PAHO chronic care model (CCM). The Strategic Plan considers that the Project also “uses” this model (p.36) and states it intention to “Integrate CCM used in rDFC project into Wellness Centers of Excellence” (p.67). This Plan and the redeployment of a coordinator for CNCD are extremely important developments and lay the groundwork for further collaboration.
2. Other specific measures by which the Ministry has embedded Project innovations:
3. Project assessment tools, indicators, practice guidelines and data spreadsheets were approved for Ministry use (April 2012)
4. The Ministry Standards and Technical Services Branch has developed a Standard Operational Procedure manual for reporting on the Guyana Diabetes and Footcare programme, including a list of indicators and the first Inspectorate visit will take place in April 2013.
5. Seven clerks hired by the Project for regional foot centres and Health Statistics Unit have been incorporated in public service.
6. The Key Opinion Leader team has come under the management of the Institute of Health Sciences Education - responsible for post-graduate medical and health education in Guyana. MoH has committed to fund the final residential weekend of the 2012-13 IIWCC.
7. Since 2010, the Ministry through its Rehabilitation Branch has purchased all plantar pressure redistribution devices for the public system
8. Ministry has achieved some success in making HbA1c testing available to PWD in Guyana through the National Public Health Reference Laboratory and in three other regions of the country as well as the main testing equipment at GPHC
9. The Ministry has solicited and received input from the Project on: the CNCD component of the Package of Publically Guaranteed Health Service; the Service Level Agreements for the Regional Health Services; the MoH budget for CNCD and the Ministry public drug formulary
10. **Main lessons learned**

The main lessons learned were the essential need for:

- Effective collaboration
- Understanding local conditions
- Engaging partners early and thoroughly
- Setting realistic objectives and targets

1. **CAGS cost-sharing obligations**

The cost-sharing obligation of CAGS was 100% in-kind, budgeted initially as $368,000 over the course of the Project. This was increased to $487,642 in a budget revision. The total in-kind amount actually contributed to April 2013 is $532,400. This is 51.6% of the total cost-shared budget. The amount is based on in-kind hours donated by CAGS volunteers ($100/hour for physicians and $50/hour for allied health) in Canada and in Guyana as well as the in-kind hours donated by the project coordinator ($100/hour).

1. **Explanation of Variances**

The total budgeted CIDA contribution was $498,925.

CIDA’s share of direct expenses is $435,639

CIDA’s share of direct expenses + overhead is $487,916

The explanation of line items with variances > $5000 or 10% is as follows:

| **Line item** | **Variance (%)**  **(minus (in red):**  **actual expense > budget)** | **Comment** |
| --- | --- | --- |
| D. In Guyana Honoraria  for trainers | $10,301 (30) | Since total workshops were reduced  the allocated funds for trainer honoraria were not used |
| E. In Guyana Rehabilitation  6 sites plus equipment | -$18,153 (-31) | Unused funds from other line items were used to purchase needed equipment |
| F1. Production costs  for training materials | $5,909 (35) | Since # workshops were reduced  allocated funds for production costs were not used |
| F2. Venue rental | -$248 (-15) | Minor amount |
| G2. Office expenses Canada | -$400 (-18) | Minor amount |
| J. Travel: Training outside  Region 4 | -$1,600 (-10) | Minor amount |
| L. Travel: Monitoring costs | $14,880 (64) | Reduced # workshops means unused  funds |
| M. Travel: Dissemination | -$1,921 (-32) | Minor amount |
| O. Staff recruitment in Guyana | -$315 (-26) | Minor amount |
| P. Gain/loss on foreign  exchange | -$1,884 (-376) | Failure to adequately understand basis of calculation of item. Budget reduced from $3000 in previous amendment. |

**Submitted May 31, 2013:**

**
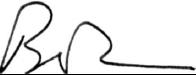
**

**Brian Ostrow MD, FRCSC**

**Project coordinator**

**Appendix A - Summary of renovation expenses**

**Appendix B - Equipment purchased**

**Appendix C - Intellectual property**

**Abbreviations:**

**BBDC – Banting and Best Diabetes Centre**

**CA – Contribution Agreement**

**CAGS – Canadian Association of General Surgeons**

**CNCD – Chronic Non-Communicable Diseases**

**CIDA – Canadian International Development Agency**

**COE – Center of Excellence**

**DFC – Diabetic Foot Centre**

**GPHC – Georgetown Public Hospital Corporation**

**HCP – Health care professional**

**HC/HP – Health centre/health post**

**IIWCC – International Interprofessional Wound Care Course**

**KOLs – Key opinion leaders**

**MoH – Ministry of Health**

**NCC – National coordinating committee**

**PAHO – Pan-American Health Organization**

**PMF – Performance Management Framework**

**PSU – Program support unit**

**PWD – persons with diabetes**

**RDFC – Regional Diabetic Foot Centre**
